# Spectroscopic Insights into the Influence of Filling Carbon Nanotubes with Atomic Nanowires for Photophysical and Photochemical Applications

**DOI:** 10.1021/acsanm.2c05266

**Published:** 2023-02-08

**Authors:** Ziyi Hu, Ben Breeze, Marc Walker, Eric Faulques, Jeremy Sloan, James Lloyd-Hughes

**Affiliations:** † Department of Physics, University of Warwick, Gibbet Hill Road, Coventry CV4 7AL, United Kingdom; ‡ Institut des Matriaux de Nantes Jean Rouxel, CNRS, University of Nantes, Nantes F-44000, France

**Keywords:** carbon nanotubes, HgTe, nanowires, Raman, ultrafast, XPS

## Abstract

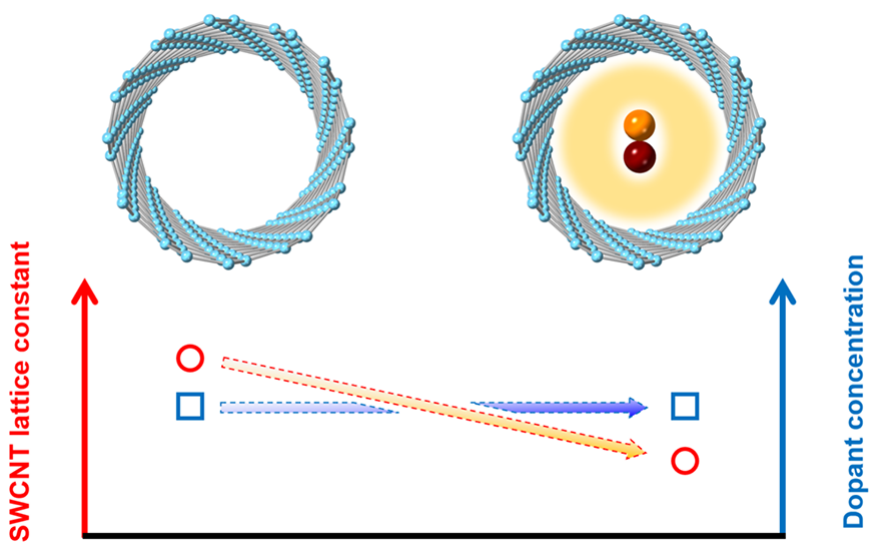

Studying the optical performance of carbon nanotubes (CNTs) filled with guest materials can reveal the fundamental photochemical nature of ultrathin one-dimensional (1D) nanosystems, which are attractive for applications including photocatalysis. Here, we report comprehensive spectroscopic studies of how infiltrated HgTe nanowires (NWs) alter the optical properties of small-diameter (*d*_t_ < 1 nm) single-walled carbon nanotubes (SWCNTs) in different environments: isolated in solution, suspended in a gelatin matrix, and heavily bundled in network-like thin films. Temperature-dependent Raman and photoluminescence measurements revealed that the HgTe NW filling can alter the stiffness of SWCNTs and therefore modify their vibrational and optical modes. Results from optical absorption and X-ray photoelectron spectroscopy demonstrated that the semiconducting HgTe NWs did not provide substantial charge transfer to or from the SWCNTs. Transient absorption spectroscopy further highlighted that the filling-induced nanotube distortion can alter the temporal evolution of excitons and their transient spectra. In contrast to previous studies on functionalized CNTs, where electronic or chemical doping often drove changes to the optical spectra, we highlight structural distortion as playing an important role.

## Introduction

Following the first reports of filling carbon nanotubes (CNTs) with guest materials 3 decades ago,^1^ there has been huge interest in the creation of extremely thin one-dimensional (1D) hybrid materials such as CNTs filled with molecules,^2−6^ other nanotubes,^7−9^ nanowires (NWs)^10−13^ or nanoclusters. One prominent emerging application is the use of CNTs and nanotube heterostructures in photocatalysis, where filled or functionalized nanotubes can boost the rate of photocatalytic redox reactions that remove pollutants, perform CO_2_ reduction, or produce hydrogen from water.^14−16^ For such applications, knowledge of the electronic and optical properties of the nanocomposite is vital, in particular because it has reported that the infiltrated material can significantly modify the electronic nature of the outer nanotubes.^17−20^ For example, filling with Te can enhance the charge-carrying capacity of boron nitride nanotubes,^12^ growing copper halide nanocrystals inside CNTs can lead to the creation of new carbon-copper energy levels,^21^ and filling with alkane can significantly enhance the tunability of the optical performance of CNTs.^22^ Further, hybrid nanotube materials,including heterostructures with BN and MoS_2_ and other nanotubes, exhibit unique properties such as unconventional Raman signatures, distinct phase transitions, and interesting charge-transfer processes between their constituents.^17−20,23,24^ Tremendous developments in liquid-phase separation techniques have enabled the purification of not only the “host” material, such as single-walled carbon nanotubes (SWCNTs),^25−28^ but also nanotube heterostructures based on their metallicity or electronic properties.^29,30^ Nanotube heterostructures are also attractive components for nanoelectronics, where they can act as functional thermoelectric, photovoltaic, or conductive elements. In order to enable applications of nanotube heterostructures in photocatalysis, often in the solution phase, and nanoelectronics, where thin film geometries are more relevant, it is important to investigate how these composites behave when embedded in different environments.

The optical and electronic properties of SWCNTs can be understood to a large degree based on the electronic properties of graphene, after accounting for the helical and rotational symmetries of the nanotube^31^ and many-body interactions.^32^ For the lowest two excitonic transitions of semiconducting SWCNTs, *S*_11_ and *S*_22_, the many-body correction is relatively small,^32^ and hence tight-binding models can accurately describe the influence of structural distortion on the excitonic transition energies.^33^ For example, previous studies discovered an excitonic energy shift of SWCNTs in response to temperature and phase changes of the suspension medium.^34,35^ It was shown that the observed experimental shifts were clearly dependent on the nanotube chiral angle and family type,^32^ with the dominant changes in optical absorption originating from uniaxial deformation. Recently, a similar evolution of the SWCNT spectra after filling with alkane molecules was observed and assumed to be the result of a change in strain along the nanotube radial direction.^20^ However, comprehensive analyses on the distortion of the SWCNT structure by spectroscopic characterizations are hindered by several factors such as doping effects^21,36^ and the variation in the dielectric constant^37^ of the filling.

Here we report an investigation of the optoelectronic properties of SWCNTs filled with HgTe NWs using a variety of spectroscopic probes. HgTe NWs provided an attractive candidate to explore the impact of nanotube distortion because of their high filling fraction, as well as the sufficiently small level of electronic charge transfer from filling to/from the SWCNTs, making it easier to determine how their optical properties varied following structural changes. Filled semiconducting SWCNTs, with predominantly small nanotubes (*d*_t_ < 1 nm), were chosen for this study: because they exhibit strong excitonic absorption and emission spectra, they are more attractive than metallic nanotubes for nanoscale optoelectronic devices. The optical properties were examined for different CNT chiralities and with different dielectric environments: isolated in solution, in a polymer matrix, or in large bundles in a thin film. On the basis of temperature-dependent photoluminescence (PL) and Raman characterizations, the presence of HgTe NWs was found to alter the excitonic and vibrational energies of the encapsulating SWCNTs in a way that depended on their chirality. In transient absorption (TA) pump–probe spectroscopy,^13^ the recombination rate of the transient exciton population was altered by HgTe NW infiltration. It was found that SWCNT species belonging to different semiconducting families [i.e. (6,5) and (7,5)] displayed opposite changes in their TA spectra, indicating the prominent effect of nanotube structure distortion following pump-induced thermal heating.

## Results and Discussion

### Nanostructure and Composition

**Figure 1:**
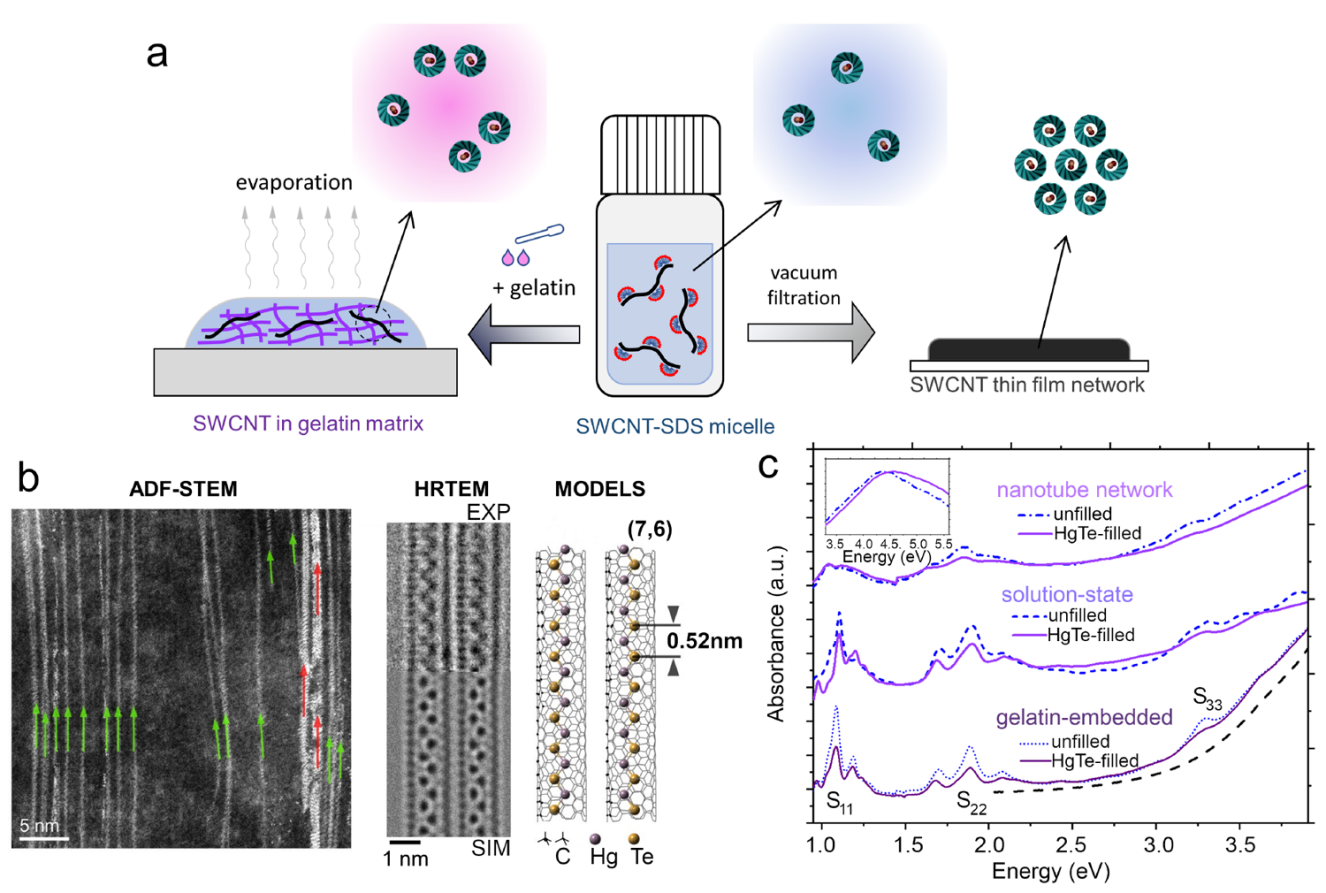
(a) Schematic diagram to depict the methods of preparing SWCNT samples in different existing states. (b) Left: ADF-STEM images of the HgTe NW-filled semiconducting SWCNTs. Green and violet arrows mark the one-atom-thick zigzag NWs and wider NWs observed from the ADF-STEM image. Middle: Experimental (top) and simulated (bottom) bright-field high-resolution TEM images of two SWCNTs filled by zigzag atomic NWs. Right: Structure model of zigzag atomic NW-filled (7,6) SWCNTs. (c) UV–vis–NIR absorption spectra of SWCNT samples in different states. The dashed curve is the rescaled absorption spectrum of a blank gelatin film. The inset shows the absorption spectra of the vacuum-filtered thin films at higher energies, which revealed the π-plasma feature.

SWCNT samples were prepared with and without infiltrated HgTe NWs in different dielectric environments, as shown in Figure 1a: solution phase, gelatin-embedded, and thin film. The gelatin-embedded material was prepared by adding a gelatin solution to the sodium dodecyl sulfate (SDS)-mediated SWCNT solution (*m*_gelatin_:*m*_SWCNT_:*m*_water_ = 1:2:10) and then drop-casting the mixed solution onto the quartz substrate. The SWCNT thin-film network was prepared based on vacuum filtration using a cellulose acetate membrane followed by transfer of the film onto the quartz substrate.

The atomic structure of HgTe-filled semiconducting SWCNTs was studied by annular-dark-field scanning transmission electron microscopy (ADF-STEM) and high-resolution transmission electron microscopy (TEM) imaging (shown in Figure 1b). Electron microscopy images evidenced that HgTe can be successfully infiltrated into narrow nanotubes (*d*_t_ < 1 nm), forming a dominant phase of zigzag-type atomic NWs with an averaged Te–Te (Hg–Hg) separation of ∼0.52 nm and a Te–Hg–Te bond angle of 110°. The scanning electron microscopy (SEM) image and atomic force microscopy (AFM) topography of vacuum-filtered SWCNT films are shown in Figures S1 and S2.

The UV–vis–near-infrared (NIR) spectra of SWCNT samples in different environments (vacuum-filtered thin film, solution state, and gelatin film) with and without HgTe infiltration are shown in Figure 1c. HgTe-infiltrated samples (solid purple lines) had absorption spectra similar to those of unfilled samples (blue dashed lines), while the morphology of the nanotubes created more significant changes. The excitonic absorption lines of thin-film-state SWCNTs (top) were markedly red-shifted and broadened compared to those of isolated solution-state (middle) and gelatin-embedded (bottom) samples. The significant nanotube bundling of SWCNTs in the vacuum-filtered film results in strong changes in the excitonic properties, such as band renormalization (a different dielectric environment) altering the excitonic energies, while ultrafast tube–tube energy transfer within bundles increases the dephasing rate,^38^ broadening the excitonic lines (most clearly seen for the *S*_11_ and *S*_22_ peaks). Stronger UV absorption in the gelatin-embedded samples arose from the gelatin matrix (long-dashed line).

**Figure 2:**
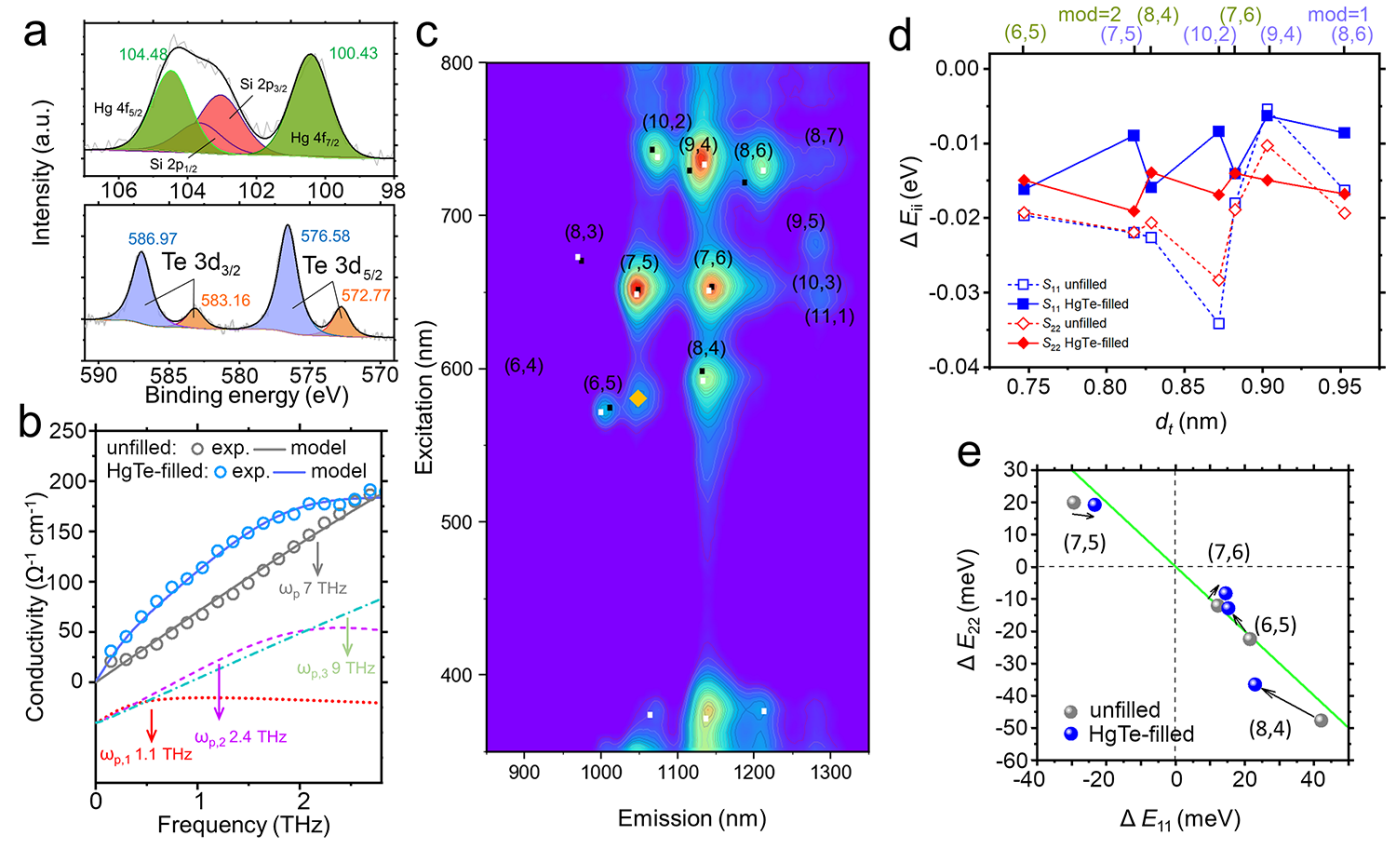
(a) XPS Hg 4f (top) and Te 3d (bottom) spectra of the vacuum-filtered HgTe-filled semiconducting SWCNT thin films. (b) THz conductivity of vacuum-filtered SWCNT films (points) and simulated values based on the surface plasmon model (lines). The dotted, dashed, and dot-dashed curves, shown offset at the bottom of figure, are the three plasma oscillators contributing to the simulated conductivity spectrum of a HgTe-filled SWCNT film (blue solid curve). The plasma resonance frequency for each oscillator is denoted in the figure. (c) PLE contour map of the HgTe-filled gelatin-embedded SWCNT film. Black and white dots represent the interband energy values obtained based on the empirical model in ref (44) and the experimental energy values of these SWCNTs in solution. The orange diamond marks an assigned phonon sideband feature of (7,5). (d) Excitonic energy shifts of various (*n*,*m*) species as a result of changes in the surrounding medium, Δ*E*_*ii*_. (e) Excitonic energy shifts induced by changes in the temperature, Δ*E*_*ii*_ = *E*_*ii*,298 K_ – *E*_*ii*,80 K_.

X-ray photoelectron spectroscopy (XPS) of the NW-filled SWCNTs is shown in Figure 2a. The reported valences of Te in crystals/alloyed compounds include Te(2−), Te(0), and Te(4+).^39−41^ On the basis of their binding energy separation, we assigned the Te peaks to a stronger Te(4+) component, with a weaker contribution from Te(0)/Te(2−).^39,41^ In the Hg 4f core-level spectrum, two peaks related to Hg were assigned to the split 4f_7/2_ and 4f_5/2_ levels (Figure 2a). The position and shape of the Hg 4f_7/2_ peak allows the Hg(2+) or Hg(0) valence states to be distinguished.^42,43^ Here, the Hg 4f_7/2_ peak can be fit by a single Gaussian peak with a central binding energy of 100.43 eV, suggesting that Hg(2+) ions were detected by XPS. Via charge balance, the Te element in HgTe NWs would then be Te(2−). The assignment of this smaller doublet in the Te 4f region is further corroborated by the composition ratio between the total Hg 4f intensity and the smaller doublet of the Te 4f region of 1:1.06. We suggest that the prominent Te(4+) feature results from residual surface oxides on extraneous material grown outside the nanotubes, such as Te^4+^O_4_^2–^. The Si 2p signal, which partially overlaps with the Hg 4f signal, originates from the carbon tape used to secure the sample.

### Conductivity

We studied the conductivity of vacuum-filtered SWCNT films by characterizing their far-infrared transmittance via terahertz time-domain spectroscopy (THz-TDS). The experimental transmittance was converted into alternating-current conductivity, the real part of which is shown in Figure 2b (points), based on the thin-film approximation.^45^ The conductivity for both unfilled and HgTe-filled SWCNT films increased toward higher frequencies. Previous results, obtained in the mid-infrared using Fourier transform infrared (FTIR) spectroscopy on similar films placed on Si substrates, indicated the presence of a strong plasmon mode around 7–9 THz for the unfilled and filled samples.^13^ Here, additional conductivity in the spectrum of the HgTe-filled SWCNT film can be seen in the 0.5–2.0 THz range. We interpreted the spectra using the axial plasmon model for a number of different plasmon frequencies, corresponding to different SWCNT lengths or bundle numbers,^46^ creating the solid curves in Figure 2b. The model was expressed as follows:
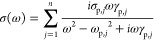
1where ω_p,*j*_ is the plasmon resonance frequency, σ_p,*j*_ is the plasmon conductivity at the resonance frequency (ω = ω_p,*j*_), and γ_p,*j*_ is the plasmon scattering rate for each of the *n* oscillators. A small number of plasmon oscillators can model the physical situation, which has a continuous distribution of CNT bundle lengths.

The fit for the unfilled sample required a single plasmon oscillator located at a relatively high frequency (solid gray line in Figure 2b). In contrast, the extra conductivity in the spectrum of the HgTe-filled SWCNT film from 0.5 to 2.0 THz was modeled by additional plasmon resonances at 1.1 and 2.4 THz (dotted and dashed lines). Consistent with our previous results from FTIR spectroscopy,^13^ HgTe filling resulted in both a broadening (the damping rate increased from 70 to 90 ps^–1^ from unfilled to filled) and a blueshifting (the central frequency increased from 7 to 9 THz) of the stronger, IR plasmon resonance peak.

The small extra optical conductivity of the HgTe@SWCNT composite may imply a weak doping of the SWCNTs after infiltration, or it may result from subtle differences in film morphology in comparison to the reference film. We return to a discussion of electronic doping later in the paper.

### PL Spectroscopy

Photoluminescence excitation (PLE) maps of unfilled and HgTe-filled gelatin-embedded SWCNT films at room temperature and 80 K are shown in Figures 2c and S3–S5, where excitonic emission from different (*n*,*m*) SWCNTs are evident. We calculated and assigned the excitonic *S*_11_ and *S*_22_ transitions using the theoretical interband transition energies introduced by a previous study:^44^
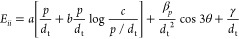
2where *p* = 1 or 2 depending on the transition order, *a*, *b*, and *c* are all constants, and *d*_t_ and θ are the diameter and chiral angle of the SWCNT. In this model, the quantum confinement of the 2D electronic structure of graphene is represented by the linear term *ap*/*d*_t_, the logarithmic term accounts for many-body electron interactions, the chiral angle-dependent term corrects for a variation with the SWCNT chirality, and γ/*d*_t_ is linked to the exciton binding energy. We found that the experimental *S*_11_ and *S*_22_ energies of most (*n*,*m*) species in the gelatin-embedded SWCNTs could be simulated using the parameters *a* = 1.049 eV, *b* = 0.456 nm, *c* = 0.795 nm^–1^, β_1_ = 0.05 nm^2^ [mod(*n*–*m*) = 1]/–0.07 nm^2^ [mod(*n*–*m*) = 2)], β_2_ = −0.19 (mod = 1)/0.14 (mod = 2), and γ = 0.305 eV. By increasing the strength of the log term from *c* = 0.795 to 0.814 nm^–1^, the recalculated energies for these small-diameter (*n*,*m*) [e.g., (6,5), (7,5), (7,6), and (10,2)] were found to be closer to the experimental energies of those in a solution state (Figure S6), indicating that encapsulation in gelatin can potentially modify the many-body Coulomb interactions within the SWCNTs.

The difference between the experimentally determined *E*_*ii*_ of solution-state and gelatin-embedded SWCNT samples, Δ*E*_*ii*_ = *E*_*ii*,gelatin_ – *E*_*ii*,solution_, is shown in Figure 2d. For the unfilled SWCNTs (dashed lines), both *S*_11_ and *S*_22_ are at higher energies in solution than in gelatin (Δ*E*_*ii*_ ≃ −20 meV) as a result of the gelatin matrix changing the many-body interactions, whereas for the filled SWCNTs (solid lines), Δ*E*_*ii*_ ≃ −10 meV. Interestingly, for the HgTe-filled SWCNTs, Δ*E*_11_ displays an observable dependence on the CNT family, with the mod 1 family having a smaller Δ*E*_11_ than mod 2 SWCNTs. Thus, when filled, changing the outer dielectric environment alters the excitonic energies in a way that depends on the CNT family. To investigate further, Δ*E*_11_ was plotted against nanotube diameter *d*_t_ (Figure 2d) and against cos 3θ and sin 3θ (Figure S7), where θ is the chiral angle of the SWCNT. No clear dependence of Δ*E*_*ii*_ on *d*_t_ or θ was discernible, suggesting that multiple parameters in eq 2 are altered simultaneously.

We now suggest a physical mechanism by which the optical properties of SWCNTs depend on the environment. In a SDS solution, a SWCNT is believed to have an outer micellar coating with a configuration depending on various factors, such as the SWCNT chirality or metallicity,^25,47^ the pH of the medium,^48^ and the lengths of SWCNTs.^49^ Presumably, immobilizing the micelle-coated SWCNTs in a gelatin matrix can result in a change in the configuration of surfactant coatings by, for example, replacing SDS at the surface with gelatin molecules. This can modify the local dielectric constant felt by charges in the SWCNTs and lead to a change in their excitonic and optical properties.

We used a temperature-dependent PLE study to further examine the excitonic properties of unfilled versus HgTe-filled SWCNTs. The temperature-induced *E*_*ii*_ shifts (Δ*E*_*ii*_ = *E*_*ii*,298 K_ – *E*_*ii*,80 K_) for four different (*n*,*m*) species in unfilled and HgTe-filled gelatin-embedded film samples are shown in Figure 2e. Taking into account a significant difference in the thermal expansion constant between the SWCNT and gelatin matrix, the observed energy shift upon cooling should result from a change of the uniaxial strain on SWCNTs,^50−52^ which is expressed as
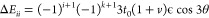
3where *i* is the transition order, *k* is the mod index of a SWCNT, which equals mod(*n*–*m*,3), and *t*_0_ ≃ 3 eV. ϵ is the uniaxial strain, while *v* ∼ 0.2 is the Poisson ratio. The experimental shifts in Figure 2e fall on the line Δ*E*_11_ = −Δ*E*_22_ (solid green line), matching the expectation from eq 3.

In previous studies, it was reported that infiltration of the CNTs by molecules can expand or distort the nanotube circumference, hence shifting the interband transition energy due to increased radial strain.^20,53,54^ The energy shift with radial strain σ varies with chiral angle θ according to^33^
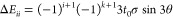
4This relationship predicts a strong family-type dependence of the energy shift. In our previous work on solution-state HgTe-infiltrated SWCNTs, a dependence of filling-induced Δ*E*_*ii*_ on *k* was seen for solution-state SWCNTs,^13^ with a generally larger Δ*E*_*ii*_ for type 1 SWCNTs such as (7,5) and (10,2) than on type 2 SWCNTs such as (8,4) and (6,5). However, no such effect can be seen in the gelatin-embedded samples (Figure 2c), suggesting that the morphology (gelatin matrix) may prevent or offset substantial radial strain.

### Raman Spectroscopy

**Figure 3:**
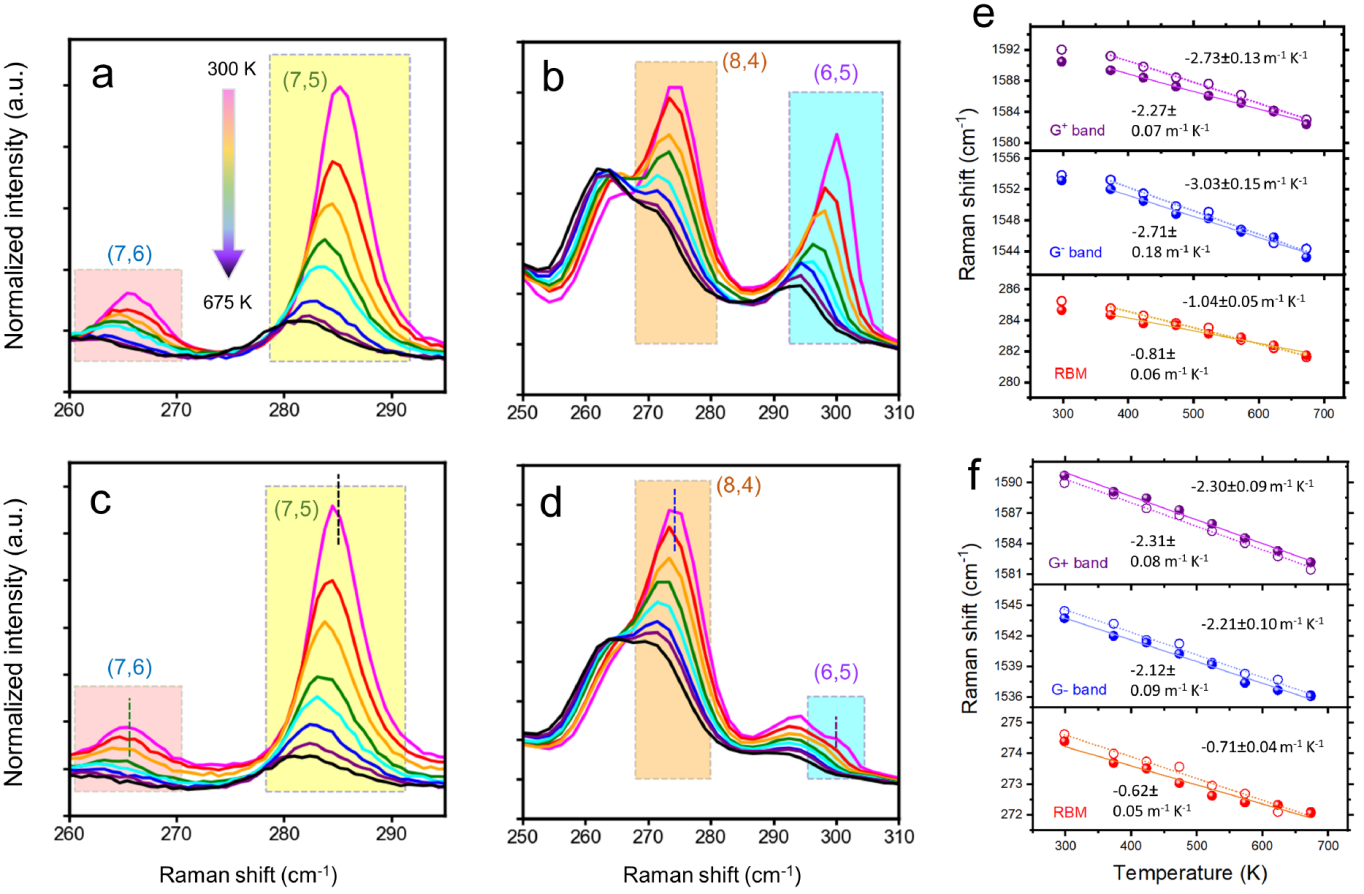
Temperature-dependent Raman spectra of the vacuum-filtered unfilled (a and b) and HgTe-filled (c and d) thin films in the RBM regime under an excitation wavelength of 660 nm (a and c) and 532 nm (b and d). The dashed vertical lines in panels c and d mark the central frequencies of the room-temperature RBM peaks for the corresponding unfilled SWCNT samples. Changes in the Raman shifts of different Raman modes for the unfilled (open circles) and HgTe-filled (solid circles) SWCNT film samples against temperature under 660 nm (e) and 532 nm (f) excitation. The dotted and solid straight lines are linear fits to the data points. The extracted thermal constants (dω_mode_/d*T*) and their errors based on a linear fit to the data are presented in the graphs.

As an alternative method to investigate how infiltration alters strain and the optical properties, we turned to resonant Raman spectroscopy.^55^ The temperature-dependent Raman spectra of vacuum-filtered SWCNT films under two different excitation wavelengths (660 and 532 nm) are shown in Figures 3 and S8–S15. The discovered radial breathing mode (RBM) signatures (Figure 3a–d) revealed that different (*n*,*m*) species were resonantly excited under the two wavelengths. Most of the Raman modes were found to display a decreased intensity, increased line-width broadening, and lower Raman shifts with a rise in temperature. Such a spectral evolution at higher temperatures has been reported in a previous work where both semiconducting and metallic SWCNTs were investigated^56^ and can be attributed to the faster dephasing of the excitonic state at elevated temperatures.

The spectral shifts of the various Raman modes with temperature were then studied (Figure 3e,f). The observed softening of both the radial and tangential frequencies at higher temperature has been previously attributed to a weakening of the intertubular and intratubular bonds.^55^ The experimental data points were well fitted by straight lines, allowing the temperature coefficient (dω_mode_/d*T*) to be determined. The temperature coefficients measured were chirality-dependent: for instance, the RBM of unfilled (7,5) SWCNTs (seen under 660 nm) had dω_mode_/d*T* = −1.04 ± 0.05 m^–1^ K^–1^, while unfilled (8,4) SWCNTs had dω_mode_/d*T* = −0.71 ± 0.04 m^–1^K^–1^. The G modes also had larger temperature coefficients for unfilled (7,5) and (7,6) SWCNTs (Figure 3e) than for unfilled (8,4) and (6,5) SWCNTs (Figure 3f).

Under 660 and 532 nm excitations, a notable reduction in the temperature coefficients after HgTe filling was found for the RBM modes (Figure 3e,f). The temperature coefficients for the G^+^ mode also reduced after filling under 660 nm excitation [i.e. probing predominantly (7,5) SWCNTs ], while under 532 nm excitation [i.e. probing (8,4) and (6,5) SWCNTs], the G modes appeared to be unaffected. For SWCNTs forming into thick bundles, as in the thin-film morphology in this Raman study, an increased temperature is thought to weaken both the intratube bonds and the intertube van der Waals forces, redshifting the RBM and G modes, whereas thermal expansion in the radial direction contributes less than 10% of the redshift.^55^ Hence, one hypothesis consistent with the above experimental results is that, while HgTe NW filling may marginally increase the diameter of the SWCNT, accounting for the lower RBM frequencies after filling, the lower temperature coefficient may result from modified force constants for the intratube or intertube bonds.^55^

Additional Raman modes were observed at Raman shifts higher than those for the G modes (Figures S13 and S15), namely, the two double-resonance phonon modes, denoted as M and iTOLA (in-plane transverse optical, longitudinal acoustic). The M mode is the predicted overtone of the out-of-plane transverse optical (oTO) mode, while iTOLA is a combination of one phonon from the in-plane TO branch and one phonon from the longitudinal acoustic (LA) branch.^57^ The M modes determined under 660 nm excitation could be deconvoluted into a lower-frequency component (∼1720 cm^–1^, referred to as the M– branch) and a higher-frequency component (∼1755 cm^–1^, referred to as the M+ branch), meaning that a phonon scattering process satisfying both |*q*| ≈ 0 and |*q*| ≈ 2|*k*| was achieved.^57^ The iTOLA modes detected under 532 nm excitation (Figure S15) displayed a higher asymmetric nature than those under 660 nm excitation (Figure S13) and were deconvoluted into a lower-frequency line (at ∼1920 cm^–1^) and one or few higher-frequency component(s), which can be explained by the emergence of additional vibrational modes such as LOLA (longitudinal optical, longitudinal acoustic).^58^ A change in the temperature coefficients of the D, M, and iTOLA modes was also detected after HgTe filling, showing a tendency (Figures S12 and S14) consistent with the above results on the G and RBM modes. Alongside the results from temperature-dependent PL, the experimental evidence is that strain due to NW filling can impact different types of vibrational motion (i.e., radial breathing and C–C bond stretching and bending), modifying the intratube and intertube bond strengths.

### Electron Doping

In this section, we return to the discussion of possible electron transfer between the HgTe and SWCNTs, which might dope the SWCNTs, which was discussed above with regard to the THz conductivity spectra. The slight redshifts of RBM and the G, D, and M bands of the vacuum-filtered HgTe-filled SWCNT films under certain excitation wavelengths [660 nm (Figures 3a and S10 and S13) and 561 nm (Figures S16 and S17) excitation] can be interpreted as a weak electronic doping of the SWCNT but may also result from a mechanical effect created by HgTe infiltration, as discussed in the previous section. Hence, in this section, we discuss electronic doping in more detail.

The level of electron doping can be estimated from the decrease in the integrated area of the excitonic absorption peak.^59,60^ In the UV–vis–NIR absorption spectra of solution-state and gelatin-embedded SWCNT samples, the two main *S*_11_ absorption peaks, assigned to the excitonic features of (7,5) and (7,6)/(8,4) SWCNTs, were suppressed in strength after HgTe filling (Figure 1c). This suggests that filling caused a small depopulation of the valence band (p-type doping) or a small population increase in the conduction band (n-type doping). The electronic influence of HgTe NW infiltration was further investigated via XPS, which has been widely used to study doping in other 1D NW/SWCNT composites. For example, XPS and ultraviolet photoelectron spectroscopy (UPS) showed that metal halides (CuX^21^ and AgX,^36^ where X = Cl, Br, or I) and metals (Ni^61^) are strong dopants, as evidenced via an observably blue-shifted sp^2^ C peak (graphene lattice π peak), attributed to charge transfer between NW and CNT. Here, for the SWCNTs filled with HgTe NWs, in contrast, there was nearly no spectral shift of the sp^2^ C 1s peak (Figure S18), which is consistent with there being little substantial doping created by the HgTe NWs. Thus, we conclude that methods that are sensitive to both structural or electronic doping changes (e.g., Raman and PL) are dominated by structural changes in this case, whereas techniques such as THz spectroscopy, which probe electronic properties only, can better assess subtle changes in conductivity.

### Ultrafast Dynamics

**Figure 4:**
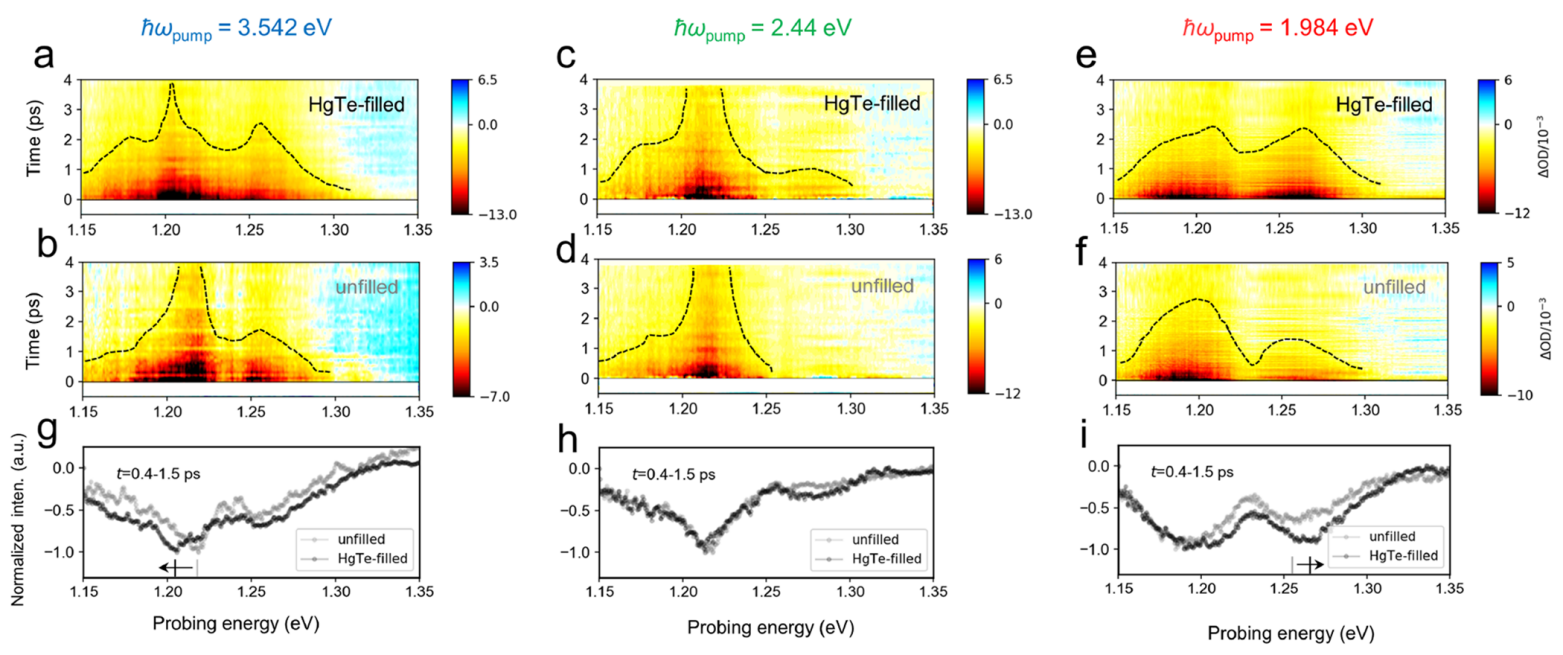
(a–f) Heatmaps of the differential transmission signals [ΔOD(*t*)/ΔOD_0_] of the solution-state SWCNTs against probe energies (1.15–1.35 eV) and pump–probe delays at various pump energies. (g–i) Corresponding spectra averaged over a time window from 0.4 to 1.5 ps. The dashed curves were added in parts a–f to indicate the spectral profiles of the GSB features. The vertical lines and arrows in parts g and i indicate the spectral shifts of GSB for (7,5) and (6,5), respectively.

Finally, TA spectroscopy was carried out across the NIR and visible ranges on samples suspended in solutions or in gelatin matrices, in order to study the relaxation dynamics of the photoinduced product (excitons and free carriers) and to act as a more sensitive probe of the excitonic states. In our previous work, we investigated exciton dynamics in the early time regime (<5 ps), where exciton–exciton annihilation (Auger) effects were prominent.^13^ In this work, we examined data obtained at later pump–probe delay times (up to 100 ps) in order to explore slower dynamical processes and investigated the influence of the surrounding environment. From the TA characteristics of solution-state SWCNTs with a pump photon energy of 3.542 eV (350 nm), close to the *S*_33_ resonance, two main ground-state bleach (GSB) peaks were evident at ∼1.25 and ∼1.2 eV (Figure 4a,b). These can be assigned to the *S*_11_ absorption features of (6,5) and (7,5) SWCNTs, respectively, while the shoulder at ∼1.18 eV on the lower-energy side of (7,5) can be attributed to *S*_11_ from a small amount of (10,2) SWCNTs. The transient spectrum was modified by HgTe filling, as witnessed by the dashed lines in Figure 4a,b (contours of constant ΔOD) and by the differing optical density change reported in Figure 4g.

**Figure 5:**
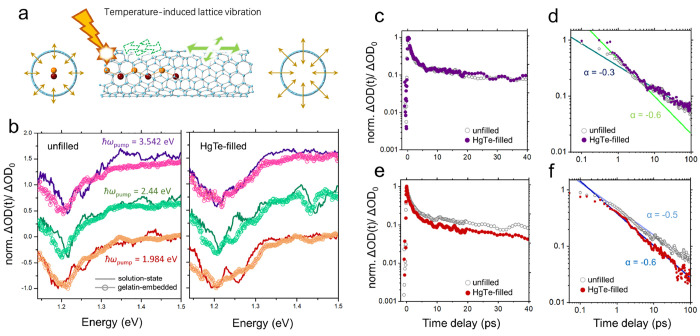
(a) Schematic showing that the suppression of in-plane (stretching and bending) and out-of-plane (radial breathing) vibrational movements due to SWCNT filling results in an increased stiffness of the nanotube upon photoexcitation. The proposed mechanism is based on the assumption that lattice vibrations along both the in-plane and out-of-plane directions are unrestricted in the liquid environment. (b) TA spectra of SWCNTs in different states averaged over a time window from 0.4 to 0.9 ps with different pump energies. All spectra were normalized to the minimum values of the corresponding GSB feature located at around 1.2 eV. Representative semilog and log–log TA kinetic curves of gelatin-embedded SWCNT films at a pump energy of (c and d) 3.542 eV and (e and f) 2.44 eV. The straight lines in panels d and f are the power-law fits [*n*(*t*) ∝*t*^α^] to the experimental data.

We then obtained TA spectra and dynamics for different excitation conditions: at 2.44 eV (508 nm) and 1.984 eV (625 nm), in the e–h pair continuum of the *S*_22_ interband transitions for all of the SWCNTs studied (Figure 4c). The discovered GSB signatures had different relative weights under different pump energies: the absorption change from (6,5) SWCNTs (around 1.25 eV) was relatively weaker at 2.44 eV excitation and was stronger for the other pump wavelengths. Interestingly, the sign of the photoinduced shifts in *S*_11_ seems to change with the SWCNT family type: type 1 (7,5) and (10,2) displayed a red-shifted *S*_11_ peak (Figure 4g), while type 2 (6,5) showed a blueshift (Figure 4i). Because the pump fluences for measurements on the unfilled and HgTe-filled SWCNTs were the same and the molarities of the nanotubes in the unfilled and HgTe-filled samples were similar (see the Supporting Information for an estimation of the molar concentrations), the shifts should not be a result of biexciton or trion generation on the *S*_11_ manifold.^62,63^ An increase in the local dielectric constant felt by SWCNTs has been reported to lead to a decreased exciton binding energy,^64^ which would create a blueshift in *S*_11_, as found after HgTe infiltration for (6,5) SWCNTs but not for (7,5). Instead, the family-dependent shifts observed in the TA spectra may result from a photothermal effect. Referring back to Figure 2e and eq 3, an elevated lattice temperature in thermal equilibrium can create either blueshifts or redshifts in *S*_11_, where Δ*E*_11_ changes sign with *k*, i.e., is opposite for (7,5) and (6,5) SWCNTs. Pulsed excitation injects energy into the electronic system, which rapidly transfers to the lattice via electron–phonon coupling. This can occur rapidly via Auger processes in HgTe-filled SWCNTs, as we previously reported.^13^ Here, however, we suggest that the family-dependent shifts in the excitonic energies seen in the experiment may be linked to modified uniaxial or radial strain coefficients in line with eqs 3 and 4. A schematic of the processes involved is presented in Figure 5a.

Compared to the TA spectra of solution-state SWCNTs, those of gelatin-embedded SWCNTs were smeared out further in energy (Figures 5b and S19), potentially as a result of inhomogeneity and/or faster dephasing caused by variations in the local dielectric function. One important consequence of this may be that the *S*_11_ excitonic states could become more similar to higher-order states (e.g., *S*_33_ and *S*_44_), where the photoexcited e–h pairs behave more like free charge carriers.

Turning now to consider *S*_11_ excitonic recombination processes, we report in Figure 5c–f the decay dynamics for (7,5) in the gelatin-embedded films under pump energies of 3.54 eV (Figure 5c,d) and 2.44 eV (Figure 5e,f). On the semilog axes (Figure 5c,e), a prominent nonlinearity in the decay curves can be observed at early pump–probe delays (<5 ps), indicating the occurrence of Auger-type nonradiative relaxation, as expected at the excitation fluences used (over 1000 excitons per nanotube). This feature is further evident in the log–log axes (Figure 5d) as the steeper slope at early times. A log–log scale further allows the reactivity of the excitons to be investigated.^65^ At the pump energy of 3.542 eV, excitons displayed a remarkable two-stage behavior because the dynamic curves for both unfilled and HgTe-filled SWCNTs followed a power-law relationship [*n*(*t*) ∝ *t*^α^] with α ≃ −0.6 before 2 ps and α ≃ −0.3 afterward (Figure 5d). This is an indicator that the excitons were more reactive in the few picoseconds after excitation due to a large amount of them being generated on a single nanotube at the applied pump fluence.^65,66^ The results also suggest that the slow lifetime component relates to diffusive excitonic motion, where excitons migrate along the SWCNTs before recombining. In contrast, when the *S*_11_ excitons were generated with lower pump photon energy (2.44 eV), the reaction kinetics were less strongly time-dependent but showed a clearer variation with HgTe filling (Figure 5f). The larger exponent (α ≃ −0.6) for HgTe-filled SWCNTs illustrates boosted recombination pathways, which may result from coupling between the electronic or vibrational systems of the NWs and SWCNTs.

## Conclusion

In this paper, we presented a detailed spectroscopic characterization of zigzag atomic chains of HgTe encapsulated within narrow-diameter SWCNTs. We studied the influence of HgTe infiltration on the optical properties of SWCNT films in a variety of different environments (solution state, embedded in gelatin, and thin film). By comparing the PL features of gelatin-embedded nanotube samples at room temperature (300 K) and at 80 K, we showed that NW filling reduces the temperature-dependent shifts for various (*n*,*m*) species, suggesting a suppressed uniaxial strain constant. Subsequent temperature-dependent Raman measurements provided evidence that the thermal coefficients of various vibrational modes (e.g., RBM mode, G mode, and double-resonance modes such as the M and iTOLA modes) decreased as a result of NW infiltration, indicating that strain can affect both in-plane and out-of-plane (radial breathing) C–C vibrational motion. These results suggest that filling with NWs alters the intratube bond stiffness of the SWCNTs, as previously reported, as well as the intertube interactions within SWCNT bundles. Here, the lower thermal coefficient after filling demonstrated that the C–C bond length changed less rapidly with temperature. This may be useful in photocatalytic applications because the electronic properties and reaction rates may be less temperature-dependent. Changes to the intertube interactions are particularly important for conductive thin-film applications of SWCNT heterostructures: if the filling modifies the intertube van der Waals interactions, the bundle density and conductivity of thin-film networks can change. Indeed, the modified THz conductivity spectra suggest that this may have occurred.

Characterizations based on optical absorption in a wide range of wavelengths, and via XPS, demonstrated that HgTe NWs had a weak doping effect on the surrounding nanotubes. This offers evidence that structural distortion, rather than direct electronic interactions (e.g., doping), controlled the spectroscopic performance of small-sized SWCNTs after infiltration. TA spectroscopy revealed a temporal evolution of exciton features depending on the SWCNT type, which features the effect of nanotube distortion under light-induced heating.

Our experimental studies demonstrate that infiltration by atomic NWs can be a novel way to control both the equilibrium-state and time-dependent properties of CNTs. This could pave the way for a comprehensive understanding of the interactions within ultrathin 1D heterostructures. In particular, for applications involving semiconducting nanotubes (e.g., photocatalysis, transistors, and photovoltaics), our results identify the important role of the filling in modifying the SWCNT structure and tube–tube interactions.

## Methods

### Materials

SWCNTs produced by CoMoCAT (ref no. 775533) were used as the raw CNT product. Sodium dodecyl sulfate (SDS; ACS reagent, ≥99.0%, Sigma-Aldrich) was used as the surfactant to produce a micelle coating and isolate CNTs. Mercury telluride (HgTe; 99%, Alfa Aesar) was used as the filling material. Hydrogels made from a cross-linked copolymer of allyldextran and *N*,*N*′-methylenebis(acrylamide) (Sephacryl S-200, GE Healthcare) were applied for the gel column chromotography experiment. Gelatin was applied as the polymer matrix for preparing gelatin-embedded SWCNT films.

### Synthesis and Purification of Materials

Processes of SWCNT filling and purification based on the gel column chromatography method were detailed in our previous work.^13^ The extracted semiconducting SWCNTs with a dominance of small nanotubes (*d*_t_ < 1 nm) were chosen for the study here. Gelatin-embedded SWCNT films were made by drop-casting a mixture of gelatin, SWCNT dispersion, and deionized water (mass ratio *m*_gelatin_:*m*_SWCNT_:*m*_water_ = 1:2:10) onto the quartz substrate and leaving it to dry at room temperature for 12 h. SWCNT thin films were prepared based on the vacuum filtration method using the cellulose filter membrane (0.2 μm, Merck). For optical measurements, the films were transferred onto the quartz substrate with membranes dissolved by acetone.

### SEM and STEM Imaging

SEM imaging was conducted based on a Zeiss Gemini microscope, which was operated under an extremely low electron voltage (0.3 kV) to avoid the charging effect. TEM and ADF-STEM were conducted based on a doubly corrected JEOL ARM200F microscope under an acceleration voltage of 200 kV and were demonstrated not to cause serious damage to the C structure. For the ADF-STEM measurement, the pixel dwell time was set to 20 μs and the chosen camera length was 8 cm, corresponding to a collection semiangle of 35–180 mrad. During ADF-STEM characterizations, the beam current density on the fluorescence screen was about 0.2 pA cm^–1^. The SWCNT samples were loaded onto the lacey TEM grid by drop-casting. The TEM grids were baked in an vacuum oven at 100–150 °C for 12 h prior to characterization in order to remove contaminants such as hydrocarbon molecules. Image analyses were carried out in the Gatan Microscopy Suite (GMS) software.

### AFM Imaging

The sample height and morphology of the material were studied by a Bruker Dimension Icon atomic force microscope. Measurements were carried out under peak force tapping mode at a tapping rate of 2 kHz, with an Al-coated SiN cantilever (tip radius = 2–12 nm, spring constant = 40 N m^–1^, resonant frequency = ∼70 kHz, length = ∼115 μm, and width = ∼25 μm) applied as the probe.

### XPS

To carry out XPS measurements on the vacuum-filtered SWCNT thin films, they were attached to electrically conductive carbon tape and mounted onto a sample bar, before being loaded into a Kratos Axis Ultra DLD spectrometer. The load lock was pumped to below 1 × 10^–6^ mbar before sample transfer to the analysis chamber. After sample transfer, the analysis chamber had a base pressure below 1 × 10^–10^ mbar. XPS was performed with the sample illuminated using a monochromated Al Kα X-ray source (*h*ν = 1486.7 eV). The measurements were conducted at room temperature and at a takeoff angle of 90° with respect to the surface parallel. The core-level spectra were recorded using a pass energy of 20 eV (resolution of approximately 0.4 eV), from an analysis area of 300 mm × 700 mm. The work function and binding energy scale of the spectrometer were calibrated using the Fermi edge and 3d_5/2_ peak recorded from a polycrystalline Ag sample prior to commencement of the experiments. The data were analyzed in the *CasaXPS* package using Shirley backgrounds and mixed Gaussian–Lorentzian (Voigt) line shapes, with asymmetry parameters where appropriate. Due to surface charging during the experiment, the samples had to be flooded with a beam of low-energy electrons in order to keep the surface from becoming positively charged. This necessitated subsequent referencing of the binding energy scale to the C–C/C–H adventitious C component at 284.6 eV. For compositional analysis, the analyzer transmission function was determined using clean metallic foils to determine the detection efficiency across the full binding energy range.

### Steady-State Spectroscopy

UV–vis–NIR (260–1800 nm or 4.77–0.69 eV) and far-infrared (0.2–3 THz or 0.8–12.4 meV) absorbance of the material was characterized by a PerkinElmer Lambda1050 spectrometer and a THz-TDS setup where photoconductive antenna and ZnTe were served as the emitter and detection crystal, respectively.^67^

Raman measurements were carried out on Renishaw InVia Reflex (532 nm excitation), Horiba T64000 triple-stage (561 nm excitation), and Horiba LabRam HR Evolution (660 nm excitation) spectrometers. A small laser power was used to avoid sample heating. For the temperature-dependent Raman measurements, the sample was placed in a Linkam stage (THMS600), which was controlled by a Linkam T96 system. The stage chamber was purged with nitrogen prior to the temperature rising. The Raman signals were measured first at room temperature and then from 100 to 400 °C with an interval of 50 °C at a ramping rate of 40 °C min^–1^. A spectrum was acquired 2 min after the temperature reached the set value. The issue of the sample moving and defocusing during heating can be neglected because the Raman spectra were normalized to their background signal intensities afterward. All Raman modes of semiconducting SWCNT species were fitted by Lorentzian functions, while the G^–^ peak of coexisting metallic SWCNTs was instead fitted by a Breit–Wigner–Fano (BWF) line shape (Figure S17). Optimized fits to the spectrum in the G-mode regime were obtained based on the Levenberg–Marquardt method.

PL of the solution-state and gelatin-embedded SWCNTs was characterized by a Horiba Fluorolog-3 spectrometer equipped with a xenon lamp that generated a broadband white-light beam. A single-photon-counting photomultiplier-tube detector was used to detect the fluorescence signals at NIR (850–1350 nm) wavelengths. The spectrometer corrected for variations in the lamp output automatically. Fluorescence of the solution-state and gelatin-embedded SWCNTs was collected in right-angle and front-face geometries, respectively. For the contour mapping measurement, a band-pass filter with nearly 100% transmittance between 332 and 807 nm and a NIR filter were placed after the excitation grating slit and before the emission grating slit, respectively. Low-temperature PL of the gelatin-embedded SWCNT films was characterized based on a cryostat (Oxford), which allowed the temperature to go down and be stabilized at 80 K. The excitonic energies of the SWCNTs were determined by fitting the experimental PL or PLE spectra with Gaussian functions and finding the peak energies.

### TA Spectroscopy

The exciton dynamics of the SWCNT samples were examined using a TA spectrometer. Both the pump and probe beams were derived from an optical parametric amplifier (TOPAS) that was seeded with a 1 kHz, 40 fs, and 800 nm pulse generated by an amplified Ti:sapphire laser (Newport Spectra Physics Spitfire Ace PA). The pump beam was mechanically chopped at 500 Hz. Different white-light probe continuua (330–720 and 700–1100 nm) were produced from a CaF_2_ crystal pumped at 800 nm and a sapphire crystal pumped at 1300 nm, respectively. A set of neutral-density filters and narrow band-pass filters were placed in the beam path to avoid saturation of the detector while affording a broadband white-light supercontinuum. The pulse width/duration of the setup was 40 fs, which defined the resolution of the experiment. The acquired TA signals were chirp-corrected. To carry out measurements under both resonant and nonresonant conditions, pump wavelengths of 350, 508, and 625 nm were chosen. The pump fluences were aimed to be set to a pretty high level (>1 × 10^16^ photons cm^–2^) in order for different relaxation pathways to be examined. Under these sufficiently large pump fluences, the transient processes were believed to enter a nonlinear regime (saturation) due to the fluence-dependent quasi-elastic dephasing.^68^

## Supporting Information Available

Supporting electron microscopy images and additional data from PL, XPS, Raman, and TA spectroscopies (PDF)
