# Supplementary material for: Spectroscopic Insights into the Influence of Filling Carbon Nanotubes with Atomic Nanowires for Photophysical and Photochemical Applications
Source: ACS Appl Nano Mater. 2023 Feb 8;6(4):2883–93. doi: 10.1021/acsanm.2c05266 (PMC9972344; doi:10.1021/acsanm.2c05266)
Supplement: Supplementary file 1 — an2c05266_si_001.pdf [file an2c05266_si_001.pdf]

Supporting Information: Spectroscopic Insights into the Influence of Filling Carbon Nanotubes with Atomic Nanowires for Photophysical and Photochemical Applications

Ziyi Hu,[*,†] Ben Breeze,[†] Marc Walker,[†] Eric Faulques,[‡] Jeremy Sloan,[*,†] and James Lloyd-Hughes[*,†]

†*University of Warwick, Department of Physics, Gibbet Hill Road, Coventry, CV4 7AL, United Kingdom.*

‡*University of Nantes, CNRS, Institut des Matériaux de Nantes Jean Rouxel, IMN, F-44000 Nantes, France.*

E-mail: ziyi.hu@warwick.ac.uk; j.sloan@warwick.ac.uk; j.lloyd-hughes@warwick.ac.uk

Experimental Details

Determination of the Concentration of (n, m) in Solution-state Samples

The number densities of different (n, m) species were determined according to the experimental absorbance and the estimated absorption cross section that was specific to each (n, m):[S1]

$$\rho_C = \frac{2.303 A(\omega)}{l \cdot \sigma(\omega)} \tag{1}$$

Here ρ_C is the number density of carbon atoms and l is the optical path length. $A(\omega)$ and $\sigma(\omega)$ are the frequency-dependent integrated decadic absorbance and aborption cross section per carbon atom, respectively.

Knowing the number of carbon atoms per unit cell of a (n, m) (*e.g.* 365 atoms in a unit cell of (6,5) which has a length of 4.06824 nm) and their average length, the number density of (n, m) species can be obtained.

To do this, optical path length and frequency-dependent decadic absorbance of the investigated SWCNT samples are required. For the solution-state and gelatin-embedded SWCNT samples, an optical path length of 1 cm and 0.02 cm were applied, respectively. For the determination of absorbance attributed to each (n, m), contribution of π-plasma backgrounds to the S_{11} spectral regime can be neglected[S2] and no background subtraction was therefore conducted. The C atom density is then calculated based on the absorbance value $A(\omega)$ as well as the absorption cross section $\sigma(\omega)$. Assuming that the semiconducting SWCNTs have

a length of 4 microns (estimated based on the AFM result in our previous work[S3]), the number density of a (n, m) species can be calculated based on:

$$\rho_{(n,m)} = \frac{\rho_C}{n_{cell} \cdot \frac{L_{(n,m)}}{L_{cell}}} \tag{2}$$

where $\rho_{(n,m)}$ is the (n, m) number density, n_{cell} the number of carbon atom in a unit cell, $L_{(n,m)}$ and L_{cell} the lengths of a nanotube and a unit cell, respectively.

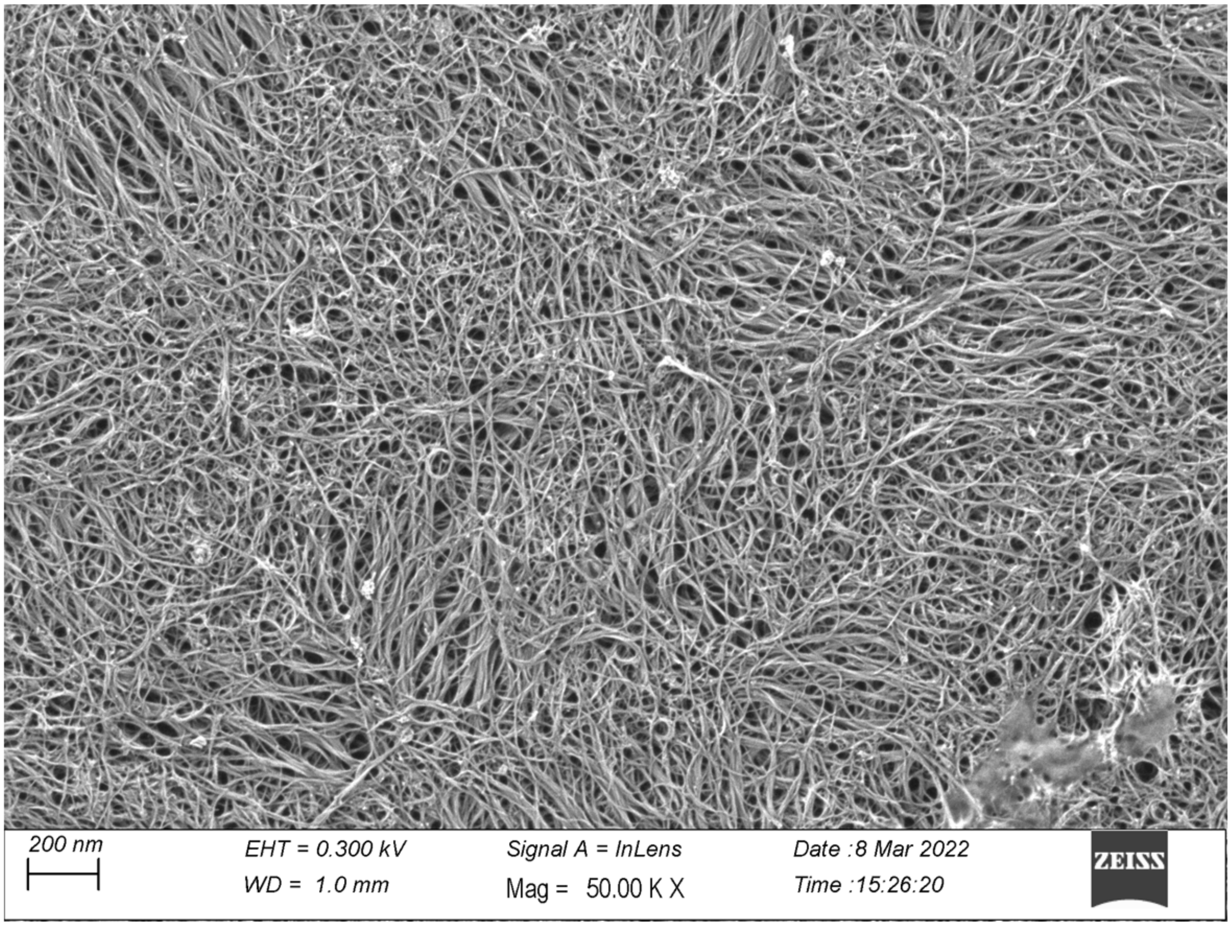

Figure S1. Low-magnification SEM image of the front surface of HgTe-filled filtrated SWCNT film.

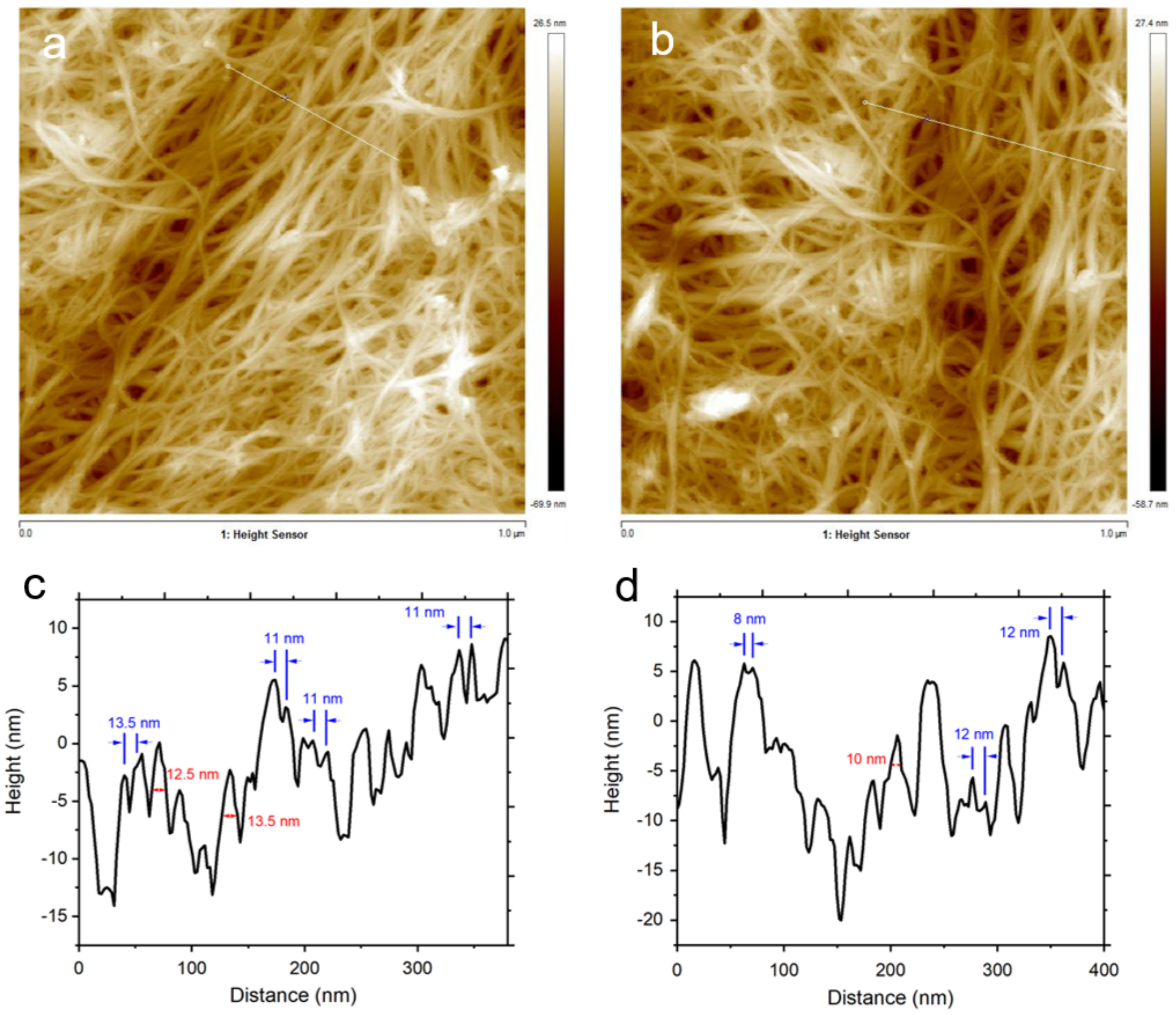

Figure S2. AFM topography of the (a) unfilled and (b) HgTe-filled filtrated films. (c,d) The corresponding cross-section line profiles. The typical thicknesses of bundles and separations between them are shown in panel (c) and (d).

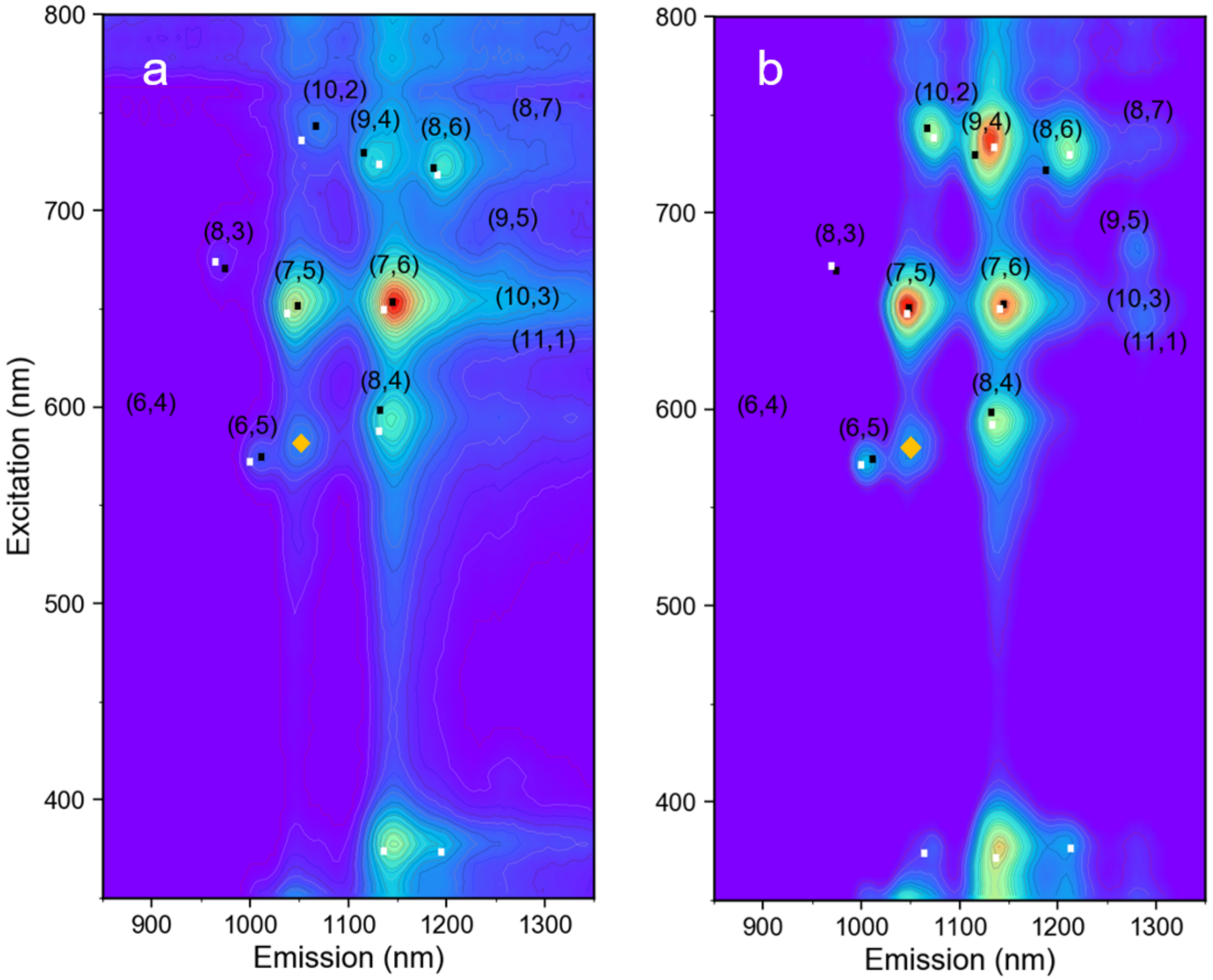

Figure S3. PLE contour maps of the (a) unfilled and (b) HgTe-filled gelatin-embedded SWCNT films. Panel (b) is the same as **Figure 2c** in the main text. Black and white dots are the interband energy values obtained based on the empirical model and the experimental energy values of these SWCNTs in a solution state. The orange diamonds are an assigned phonon sideband feature of (7,5).

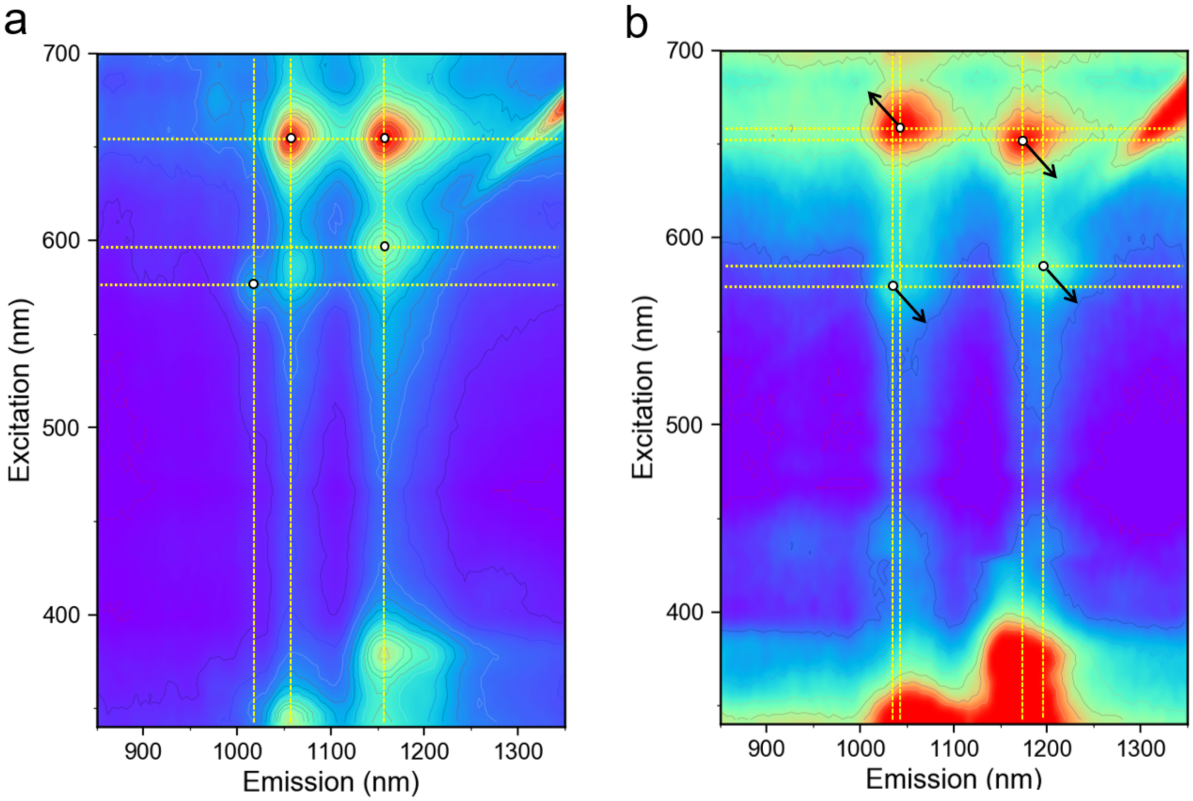

Figure S4. PLE contour maps of the gelatin-embedded unfilled SWCNT films measured at (a) 298 K and (b) 80 K. Arrows indicate the orientation of interband energy shift. The diagonal lines presenting at upper right of the contours were induced by the Rayleigh scattering.

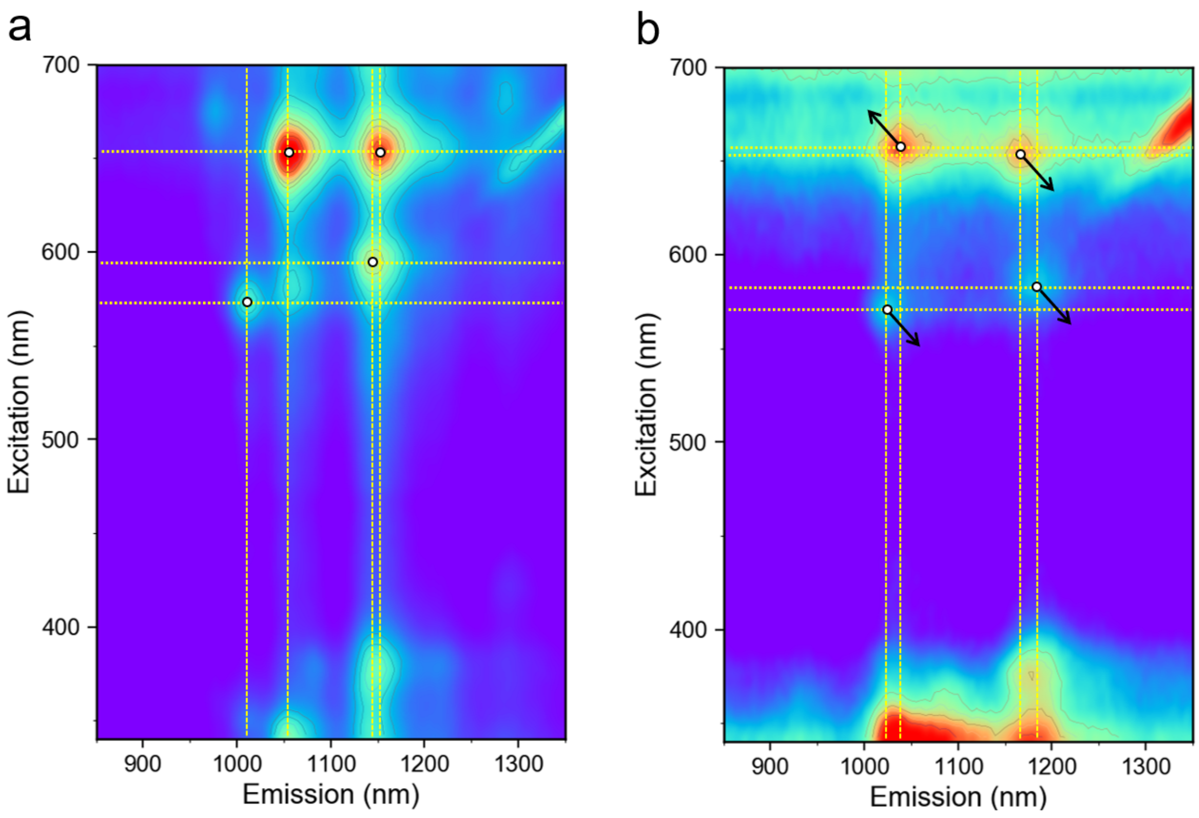

Figure S5. PLE contour maps of the gelatin-embedded HgTe-filled SWCNT films measured at (a) 298 K and (b) 80 K. Arrows indicate the orientation of interband energy shift. The diagonal lines presenting at upper right of the contours were induced by the Rayleigh scattering.

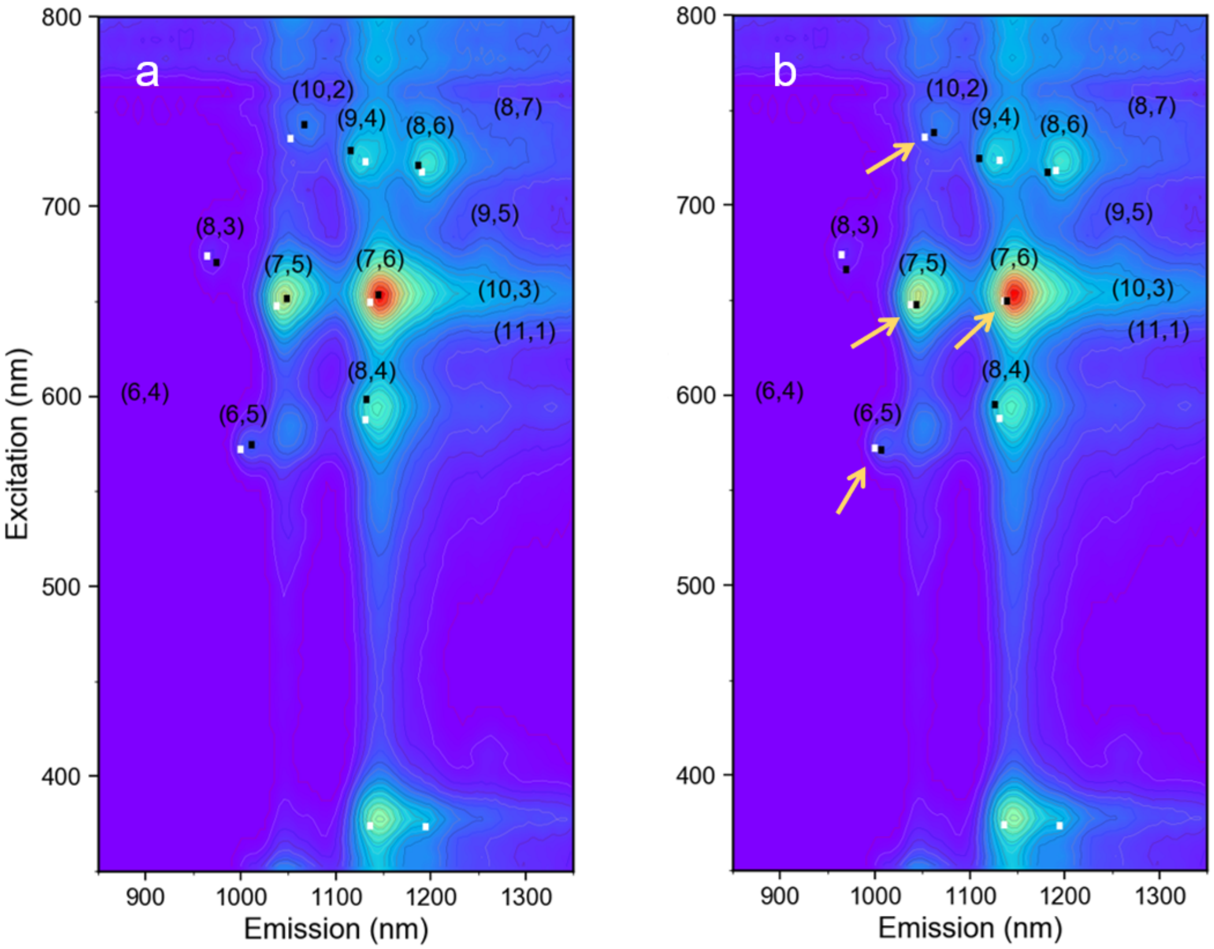

Figure S6. PLE contour maps of the gelatin-embedded unfilled SWCNT film and E_{ii} energies calculated based on different values of c parameters (black dots): (a) 0.795 nm^{-1} and (b) 0.814 nm^{-1}. White dots were E_{ii} for the corresponding SWCNTs in solution.

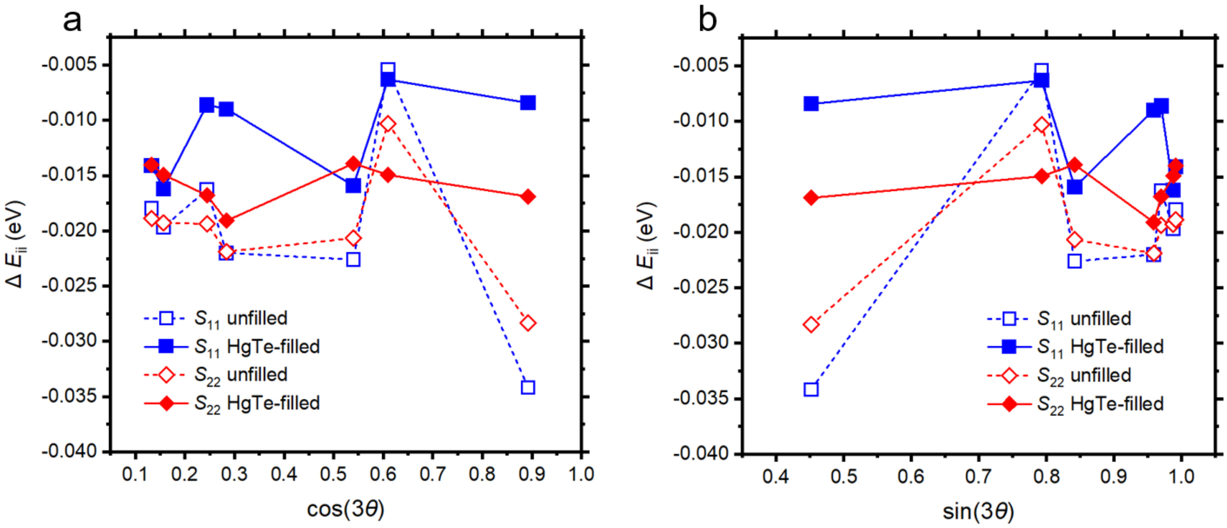

Figure S7. Energy difference (ΔE_{ii}) between the solution-state and gelatin-embedding (n, m) species determined from the PLE contour map. The ΔE_{ii} was plotted against (a) $\cos(3\theta)$ and (b) $\sin(3\theta)$, where θ is the chiral angle of (n, m).

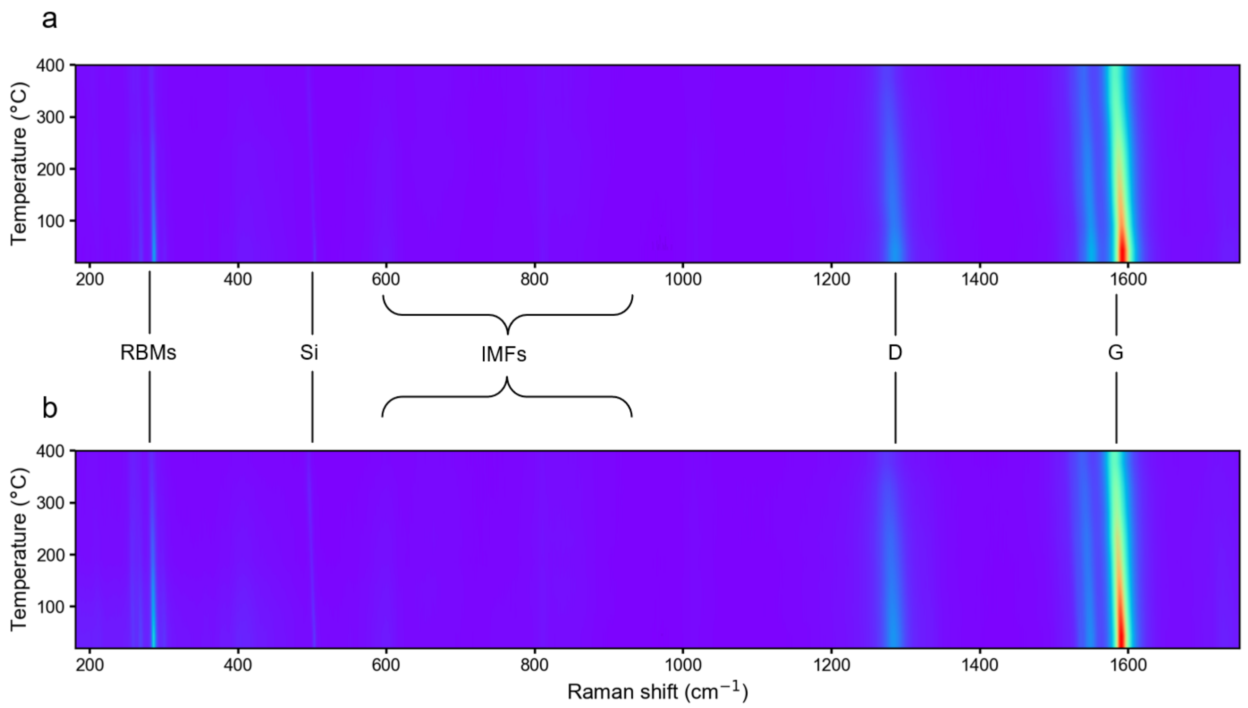

Figure S8. Heatmaps showing the evolution of Raman features for the (a) unfilled and (b) HgTe-filled filtrated SWCNT films against temperature under 660 nm excitation. 'Si' and 'IMFs' denote the features originating from the silicon substrates and intermediate-frequency Raman modes.

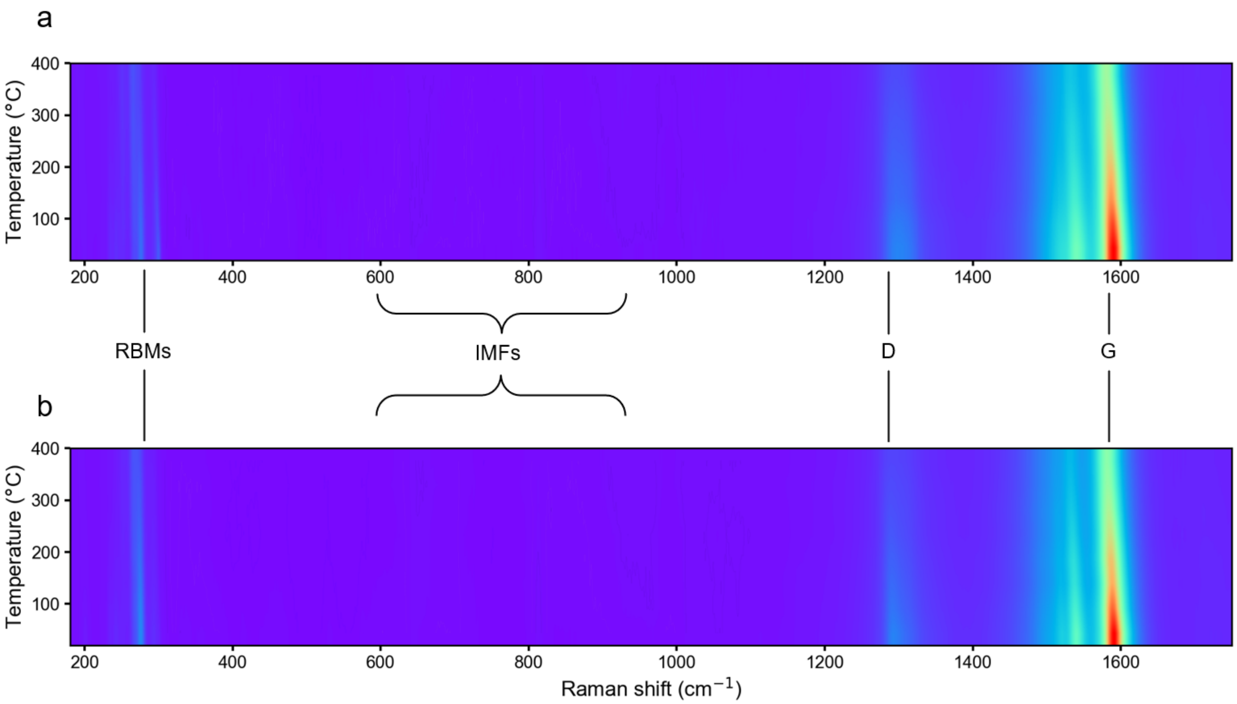

Figure S9. Heatmaps showing the evolution of Raman features for the (a) unfilled and (b) HgTe-filled filtrated SWCNT films against temperature under 532 nm excitation.

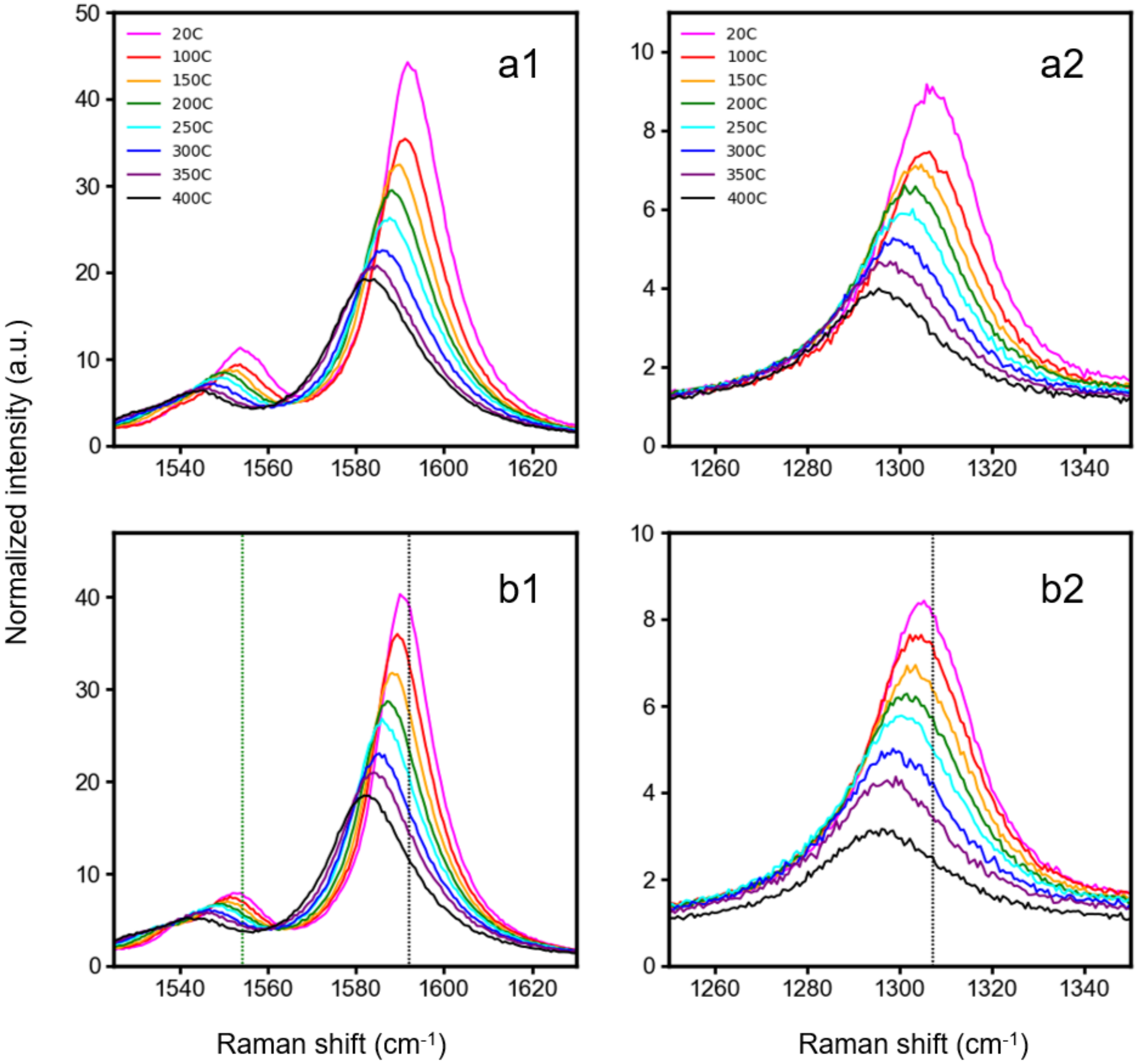

Figure S10. Temperature-dependent Raman spectra of the (a) unfilled and (b) HgTe-filled filtrated SWCNT films in the G (left) and D (right) mode regimes under 660 nm excitation.

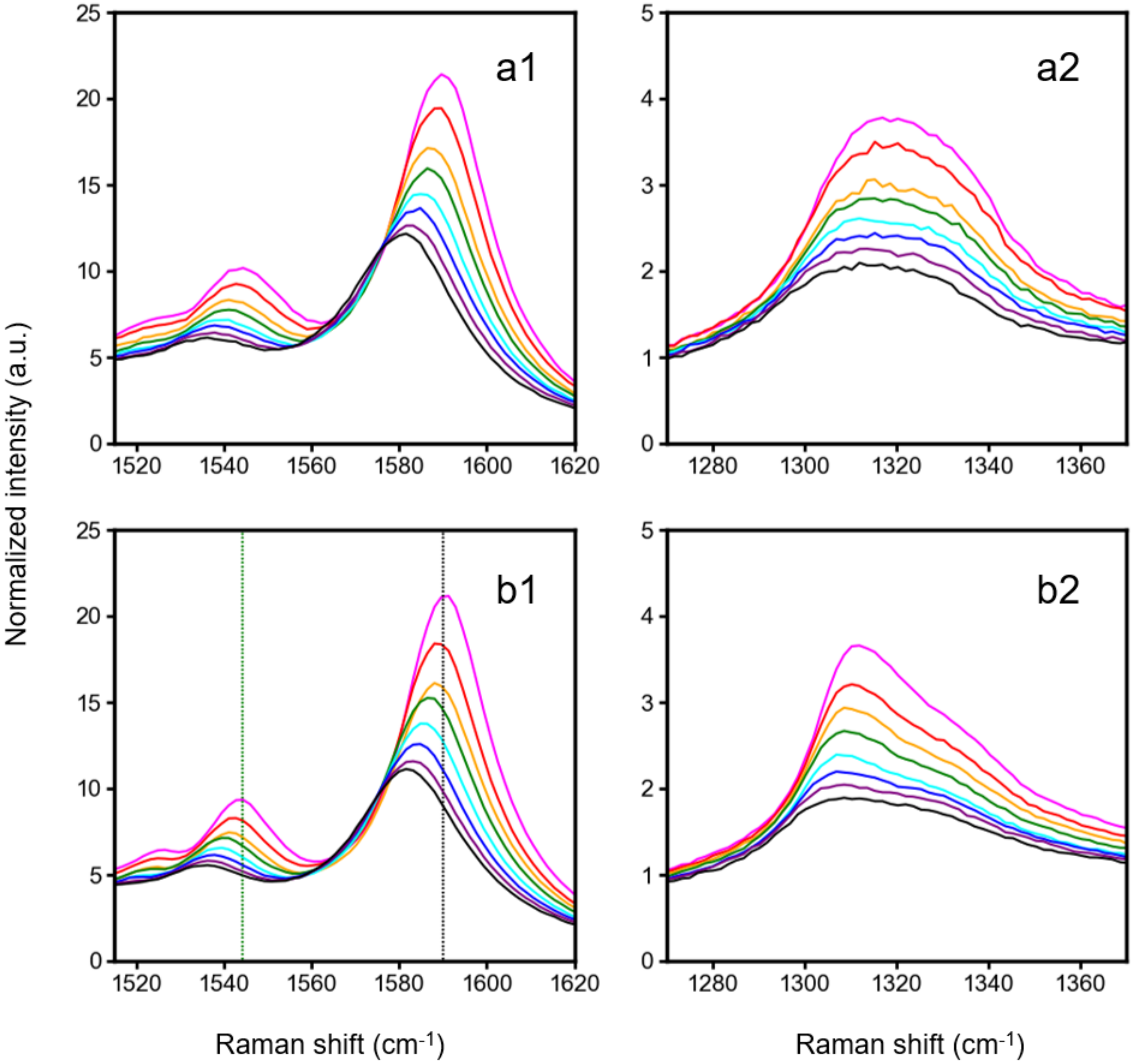

Figure S11. Temperature-dependent Raman spectra of the (a) unfilled and (b) HgTe-filled filtrated SWCNT films in the G (left) and D (right) mode regimes under 532 nm excitation.

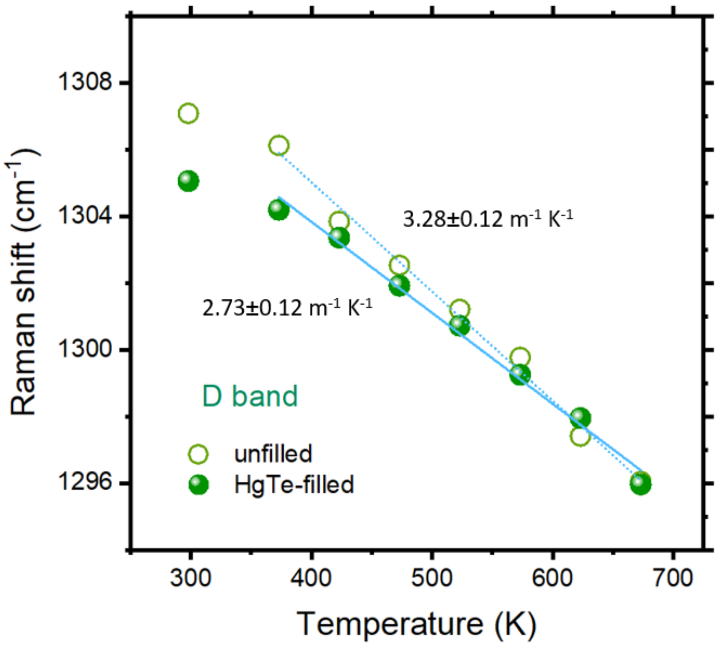

Figure S12. Changes in Raman shifts of D modes against temperature for the filtrated SWCNT films under 660 nm excitation.

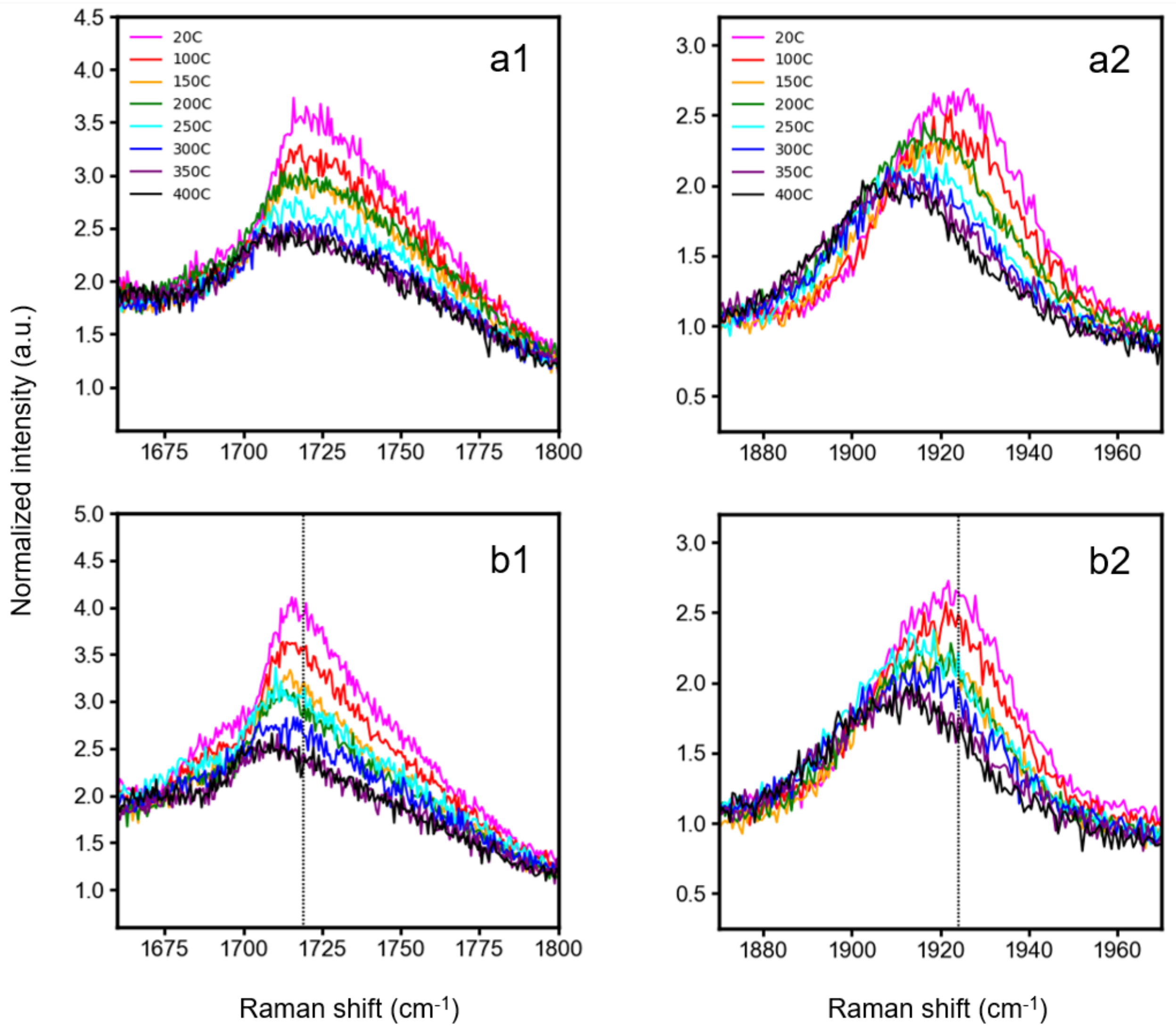

Figure S13. Temperature-dependent Raman spectra of the (a) unfilled and (b) HgTe-filled filtrated SWCNT films in the M (left) and iTOLA (right) mode regimes under 660 nm excitation.

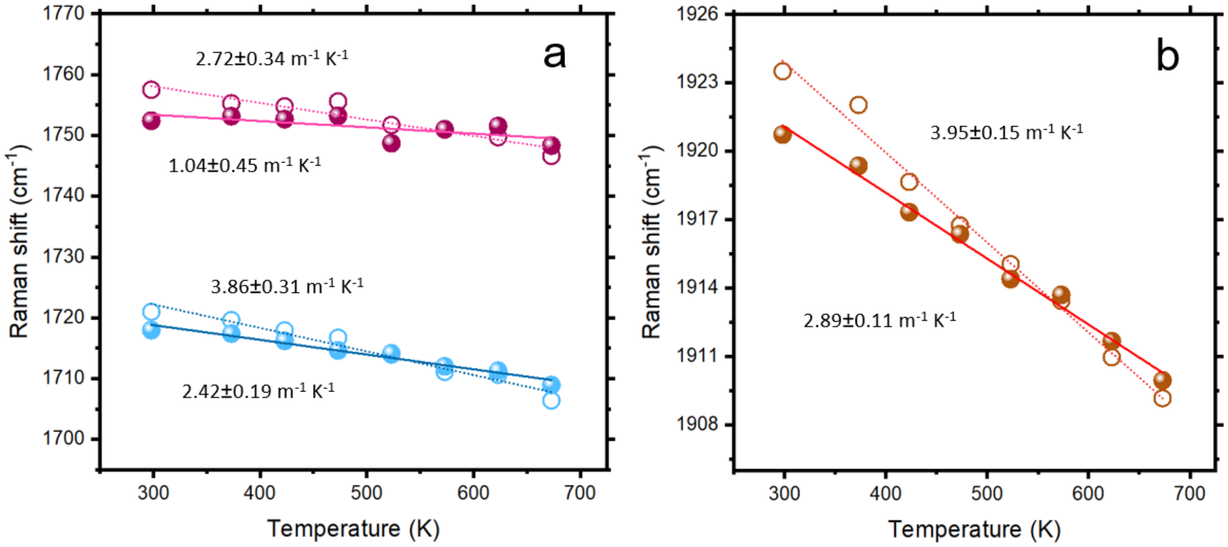

Figure S14. Changes in the Raman shifts of (a) M components and (b) iTOLA modes against temperature for the filtrated SWCNT films under 660 nm excitation.

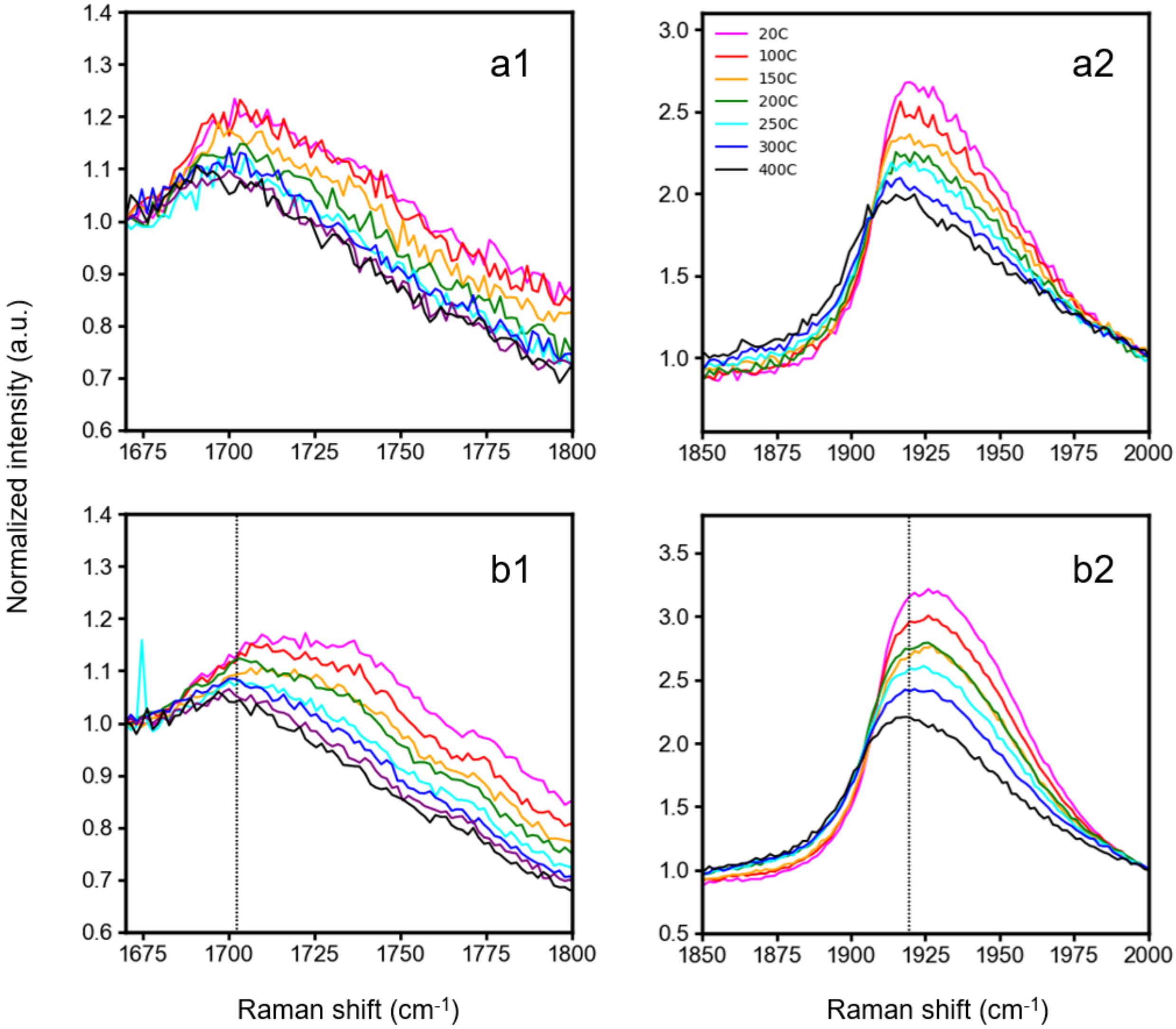

Figure S15. Temperature-dependent Raman spectra of the (a) unfilled and (b) HgTe-filled filtrated SWCNT films in the M left and iTOLA/LOLA (right) mode regimes under 532 nm excitation.

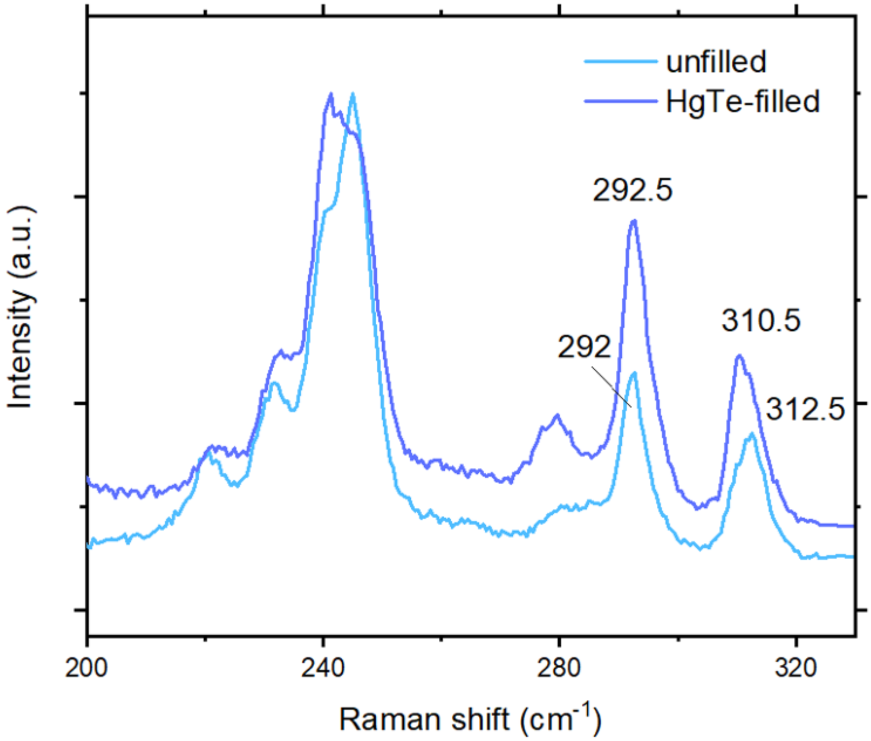

Figure S16. Room-temperature Raman spectra of the unfilled and HgTe-filled filtrated SWCNT films in the RBM regime under 561 nm excitation. The RBM peaks at 310.5 and 312.5 cm^{-1} were assigned to the signature of (6,5).

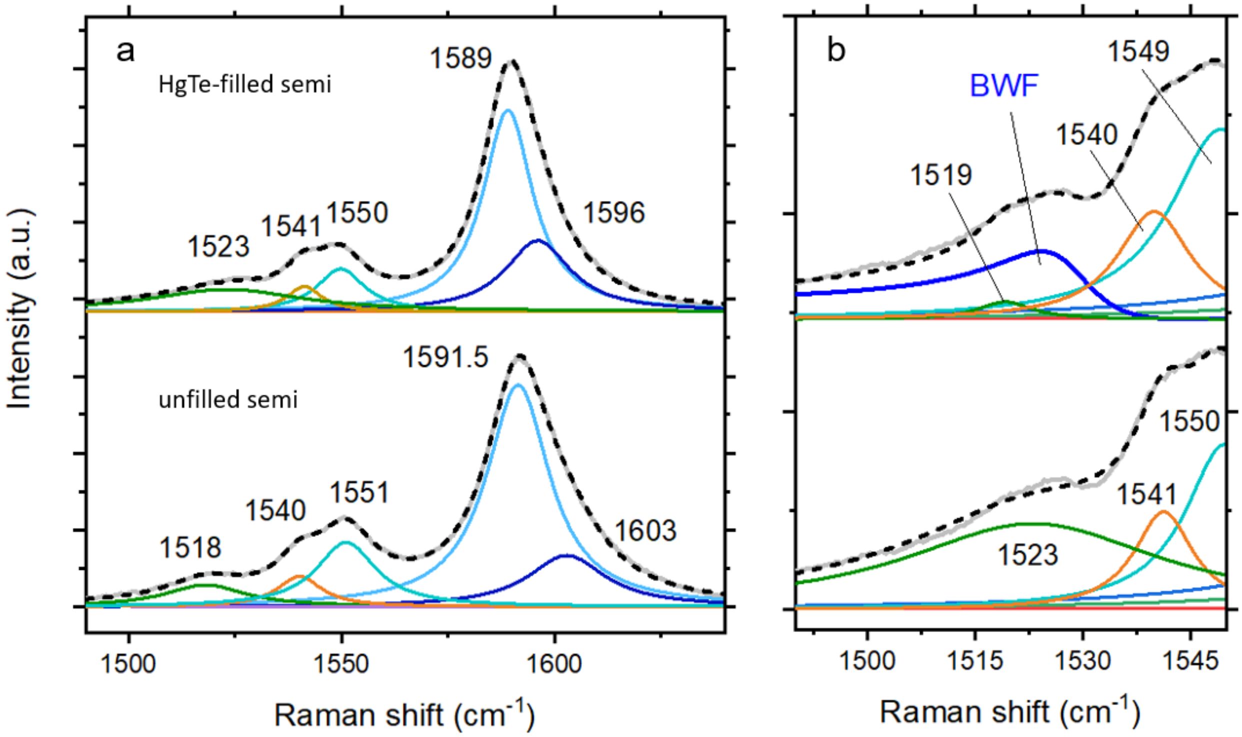

Figure S17. Room-temperature Raman spectra of the unfilled and HgTe-filled filtrated films in G mode regime under 561 nm excitation. The fine structure at $\sim 1525\,\mathrm{cm}^{-1}$ in the HgTe-filled sample spectrum can be better fitted with a BWF line than a Lorentzian (as shown at the top and bottom of panel b), indicating the existence of a slight amount of metallic SWCNTs in the sample.

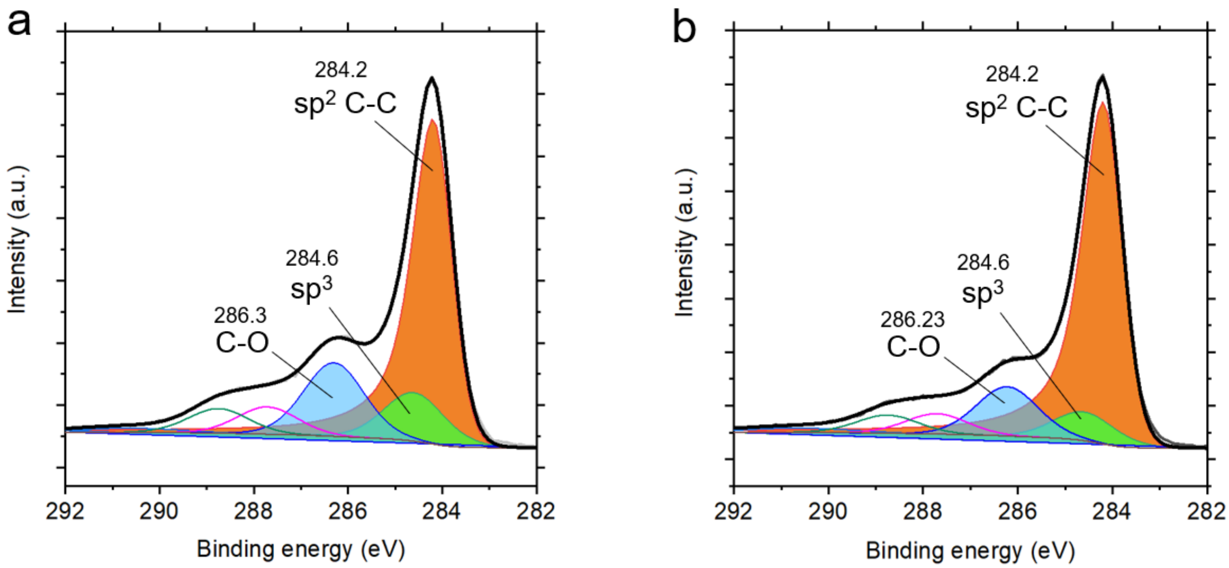

Figure S18. Deconvoluted C1s XPS spectra of the filtrated (a) unfilled and (b) HgTe-filled SWCNT thin films.

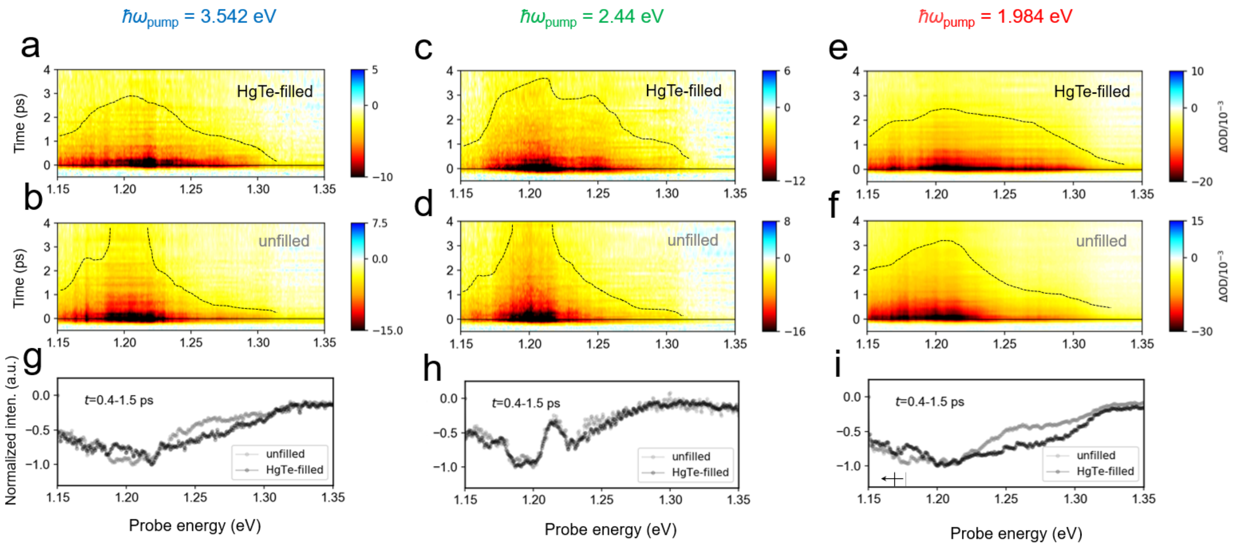

Figure S19. (a-f) Heatmaps of the differential transmission signals ($\Delta\mathrm{OD}(t)/\Delta\mathrm{OD}_0$) of gelatin-embedded SWCNT films against probe energies (1.15-1.35 eV) and pump-probe delays under various pump energies. (g-i) The corresponding spectra averaged over a time window from 0.4 to 1.5 ps. Dashed curves were added in (a-f) to indicate the spectral profiles of GSB features. Vertical lines and arrows in (i) indicate the spectral shifts of GSB attributed to (10,2).

References

(S1) Streit, J. K.; Bachilo, S. M.; Ghosh, S.; Lin, C.-W.; Weisman, R. B. Directly Measured Optical Absorption Cross Sections for Structure-Selected Single-Walled Carbon Nanotubes. *Nano Letters* **2014**, *14*, 1530–1536.

(S2) Shea, M. J.; Mehlenbacher, R. D.; Zanni, M. T.; Arnold, M. S. Experimental Measurement of the Binding Configuration and Coverage of Chirality-Sorting Polyfluorenes on Carbon Nanotubes. *The Journal of Physical Chemistry Letters* **2014**, *5*, 3742–3749.

(S3) Hu, Z.; Breeze, B.; Kashtiban, R. J.; Sloan, J.; Lloyd-Hughes, J. Zigzag HgTe Nanowires Modify the Electron–Phonon Interaction in Chirality-Refined Single-Walled Carbon Nanotubes. *ACS Nano* **2022**, *16*, 6789–6800.
